# Optoelectronic Properties in Near‐Infrared Colloidal Heterostructured Pyramidal “Giant” Core/Shell Quantum Dots

**DOI:** 10.1002/advs.201800656

**Published:** 2018-07-03

**Authors:** Xin Tong, Xiang‐Tian Kong, Chao Wang, Yufeng Zhou, Fabiola Navarro‐Pardo, David Barba, Dongling Ma, Shuhui Sun, Alexander O. Govorov, Haiguang Zhao, Zhiming M. Wang, Federico Rosei

**Affiliations:** ^1^ Institute of Fundamental and Frontier Sciences University of Electronic Science and Technology of China Chengdu 610054 P. R. China; ^2^ Institut National de la Recherche Scientifique Centre Énergie Matériaux et Télécommunications 1650 Boul. Lionel Boulet Varennes QC J3X 1S2 Canada; ^3^ Department of Physics and Astronomy Ohio University Athens OH 45701 USA; ^4^ State Key Laboratory and College of Physics Qingdao University Qingdao 266071 P. R. China

**Keywords:** near‐infrared emission, photoelectrochemical cells, pyramidal structures, quantum dots

## Abstract

Colloidal heterostructured quantum dots (QDs) are promising candidates for next‐generation optoelectronic devices. In particular, “giant” core/shell QDs (g‐QDs) can be engineered to exhibit outstanding optical properties and high chemical/photostability for the fabrication of high‐performance optoelectronic devices. Here, the synthesis of heterostructured CuInSe*_x_*S_2−_ *_x_* (CISeS)/CdSeS/CdS g‐QDs with pyramidal shape by using a facile two‐step method is reported. The CdSeS/CdS shell is demonstrated to have a pure zinc blend phase other than typical wurtzite phase. The as‐obtained heterostructured g‐QDs exhibit near‐infrared photoluminescence (PL) emission (≈830 nm) and very long PL lifetime (in the microsecond range). The pyramidal g‐QDs exhibit a quasi‐type II band structure with spatial separation of electron–hole wave function, suggesting an efficient exciton extraction and transport, which is consistent with theoretical calculations. These heterostructured g‐QDs are used as light harvesters to fabricate a photoelectrochemical cell, exhibiting a saturated photocurrent density as high as ≈5.5 mA cm^−2^ and good stability under 1 sun illumination (AM 1.5 G, 100 mW cm^−2^). These results are an important step toward using heterostructured pyramidal g‐QDs for prospective applications in solar technologies.

## 1 Introduction

Owing to features such as size‐, shape‐, and composition‐tunable optoelectronic characteristics, colloidal semiconductor quantum dots (QDs) have been widely investigated as ideal candidates for numerous optoelectronic devices including solar‐driven QDs‐sensitized solar cells (QDSCs) and photoelectrochemical (PEC) cells, light‐emitting diodes (LEDs) and luminescent solar concentrators (LSCs).^1^ For many of these potential applications, QDs are required to show efficient exciton (electron–hole pair) generation/separation and further transport with the presence of charge scavengers.^2^ However, due to the fast exciton recombination, it is still very challenging to fabricate devices with high photon‐to‐current (or fuel) conversion efficiency. For example, in PbS QDSCs, the fast exciton recombination limits the photon‐to‐power conversion efficiency (PCE), which is less than 12%, well below typical values of commercial silicon solar cells (in general around 20%) and its theoretical value (44% in QDSCs).^3^ The synthesis of heterostructured QDs and tailoring of their composition/shape are shown to be an efficient approach to control exciton and charge carrier dynamics.^4^ For instance, heterostructured tetrapod‐shaped QDs (PbSe/CdSe/CdS QDs), which are nonspherical, can favor the spatial separation of electron–hole wave functions, resulting in long radiative lifetime that facilitates charge extraction and transport.[[qv: 4a]] Other similar nonspherical colloidal systems such as dot‐in‐rod heterostructures also present highly efficient exciton extraction and transport because the photogenerated excitons in the rod region can be transported and localized in the dot region.^5^

However, there are still several significant issues in the optimization of these heterostructures (e.g., stability), which need to be addressed to improve the performance of optoelectronic devices based on such systems.^6^ For instance, in the case of heterostructures such as tetrapod‐shaped QDs and dot in rod‐structured materials, they are not stable as the shell around the core region is usually too thin (less than ≈1 nm).[[qv: 4a,5a]] Using “giant” core/shell heterostructured QDs (g‐QDs) with thick shell (thickness from 1.5 nm to tens of nm) is a promising approach to address this issue, because capping a thick inorganic shell leads to core QD's effective isolation from surrounding environment, resulting in superior thermal and photochemical/physical stability compared to core‐only/thin shell QDs.^7^ These g‐QDs systems have shown excellent optical properties such as enhanced quantum yield (QY), inhibited nonradiative Auger recombination and extended fluorescence lifetimes.^8^

The band structure of these g‐QDs systems can be tailored through a suitable modulation of their chemical composition and electronic band offset. With appropriate chemical composition, core size, and shell thickness, g‐QDs can exhibit a quasi‐type II band structure, in which the electrons are delocalized into the shell region and the holes are still confined within the core. Examples include CdSe/CdS[[qv: 8c,9]] and CuInSe_2_/CuInS_2_ g‐QDs,[[qv: 1h]] wherein the band energy levels of shell materials are wider than the core materials, but with small band offsets.[[qv: 1h,8c,9,10]] While the spherical g‐QDs can separate electrons and holes efficiently in all directions, the wave functions of electrons/holes still significantly overlap, causing fast recombination. In contrast, in some nonspherical (e.g., tetrapod and dot in rod) QDs, they show less electron–hole wave function overlap as expressed by their very long PL lifetime.[[qv: 4a,5a]] Without considering other factors which may also affect the charge separation efficiency, the smaller electron–hole wave function overlap can dramatically enhance the charge transfer rate.

Numerous kinds of g‐QDs including CdSe/CdS, CuInS_2_/ZnS, InP/CdS, PbS/CdS, CuInSe_2_/CuInS_2_, etc., have been studied in the last few years.[[qv: 1h,7b,11]] Due to their outstanding optoelectronic properties, these g‐QDs were used to fabricate both biomedical devices (e.g., biosensors) and optoelectronic devices (e.g., QDSCs, PEC cells, etc.).[[qv: 11c,12]] For example, lately, high‐efficiency and stable PEC cells were fabricated by using CdSe/CdS and CuInSe_2_/CuInS_2_ g‐QDs as light harvesters. CdSe/CdSe*_x_*S_1−_ *_x_*/CdS g‐QDs were also used for long‐term stable QDSCs with very high PCE.[[qv: 1h,12a,13]]

Although various types of g‐QDs have already been investigated and exhibit outstanding properties for optoelectronic applications, there are still several promising directions for future work: (i) Controllable synthesis of heterostructured g‐QDs with nonspherical shapes (e.g., pyramids) and fine‐control of crystal structure; (ii) the spatial distribution of electron–hole wave function in heterostructured g‐QDs with pyramidal structure; (iii) tunable near‐infrared (NIR) optical properties of heterostructured g‐QDs and their use in the NIR biomedical and optoelectronic application. Therefore, comprehensive investigations including the synthesis, optical properties, and applications of heterostructured g‐QDs with pyramidal shape and NIR absorption/emission are essential in the g‐QDs family.

Here, we describe the synthesis of CuInSe*_x_*S_2−_ *_x_* (CISeS)/CdSeS/CdS heterostructured g‐QDs with pyramidal shape and NIR emission by using a facile two‐step method. Transmission electron microscopy (TEM) images confirm the pyramidal shape with large size (up to ≈13 nm) and growth dynamics of the as‐synthesized g‐QDs. Selected area electron diffraction (SAED) and X‐ray diffraction (XRD) patterns demonstrate that the CdSeS/CdS layer shell materials crystallize in the zinc blende (ZB) structure. The optical properties show a redshift of excitonic peaks in the absorption spectra of this type of g‐QDs, which indicates the subsequent growth of alloyed CdSeS and CdS shell on CISeS core QDs. The PL spectra of g‐QDs show NIR emission (≈830 nm) and a blueshift of PL peaks in these g‐QDs demonstrates the decreased size of the QDs, which is induced by the core‐etching effect. This effect is caused by a cation exchange process in the early growth stages, which is also consistent with TEM images. The prolonged PL lifetime with increasing shell thickness indicates the quasi‐type II band structure for efficient spatial separation of electrons and holes in such pyramidal‐shaped heterostructured g‐QDs, consistently with theoretical calculations. These g‐QDs were subsequently used as light harvesters to fabricate QDSCs and QDs‐sensitized photoanode for PEC hydrogen production. The as‐fabricated QDs‐sensitized photoanode exhibits a saturated photocurrent density as high as ≈5.5 mA cm^−2^ with very good stability, comparable to the best reported QDs‐based PEC systems.^14^ Moreover, QDSCs based on these g‐QDs also exhibit good performance. These results suggest that pyramidal NIR‐emitting heterostructured CISeS/CdSeS/CdS g‐QDs are promising materials for all kinds of high‐efficiency, low‐cost, and durable photovoltaic technologies including solar‐driven PEC hydrogen production.

## 2 Results and Discussion

### 2.1 Synthesis and Structure of Heterostructured CISeS/CdSeS/CdS g‐QDs

To synthesize heterostructured CISeS/CdSeS/CdS g‐QDs with pyramidal geometry, we first prepared pyramidal‐shaped CISeS QDs via a thermal decomposition technique.^15^ The as‐synthesized CISeS QDs were then used as initial cores to grow the shell materials by using a dropwise injection of mixed Cd and S precursors (Detailed information for QDs synthesis is shown in the Supporting Information). During the growth of g‐QDs, for convenience, intermediate products formed at different growth stages were extracted and labeled as CdS#1 to CdS#9 according to the injection volumes of Cd and S precursors, as listed in Table S1 (Supporting Information).

**Figure 1:**
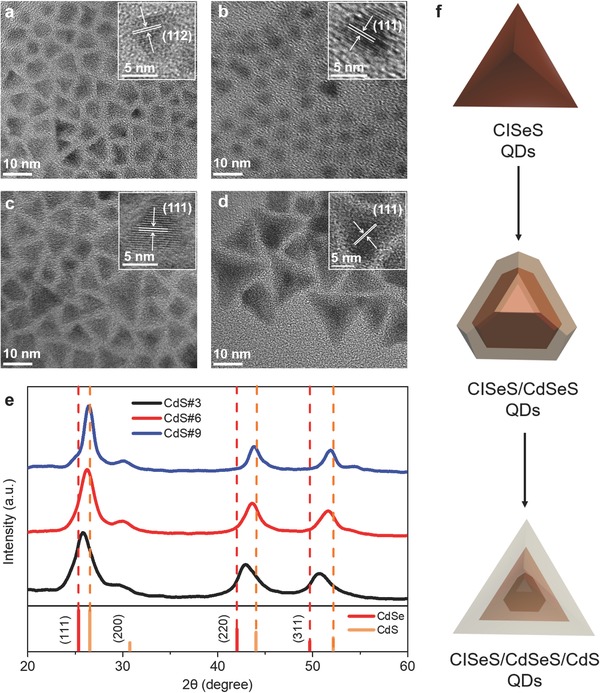
a) TEM images of CISeS with inset HRTEM images displaying (112) plane of chalcopyrite phase. TEM images of b) CdS#3 and c) CdS#6 QDs with inset HRTEM images exhibiting (111) plane of ZB phase CdSeS. d) TEM images of CdS#9 QDs with inset HRTEM images showing (111) plane of ZB phase CdS. e) XRD patterns of CdS#3, CdS#6, and CdS#9 QDs. f) Schematic diagram of growth processes and structure of heterostructured CISeS/CdSeS/CdS g‐QDs.

**Figure** **1**a shows representative TEM images of initial CISeS core QDs, which exhibit a typical pyramidal shape with average size of 5.5 ± 0.7 nm (the sizes of these QDs are defined as the height of the projected triangles^15^ and summarized in Figure S1, Supporting Information). The synthesis and morphology of CuInS_2_, CuInSe_2_, and CISeS QDs have been studied extensively in the literature.^15^, ^16^ Previous investigations confirmed that these QDs possess a pyramidal shape. We followed an approach reported in the literature^15^ and found that as‐synthesized CISeS QDs (Figure 1a) exhibit a pyramidal shape. The inset high‐resolution TEM (HRTEM) image displays a lattice spacing of 0.328 nm that is well indexed to the (112) plane of alloyed CISeS QDs with chalcopyrite phase,[[qv: 1g,17]] which is consistent with XRD and SAED patterns (Figure S2, Supporting Information) of as‐synthesized CISeS QDs.

Compared with CISeS QDs, TEM images of CdS#3 (with 2.5 mL of injected Cd/S precursors) QDs (Figure 1b) present a decreasing average size of 4.0 ± 0.3 nm and different shapes. The decreasing size of QDs is attributed to the cation exchange process following the injection of precursors at an early stage, which is consistent with previous work on similar QD systems (i.e., CuInS_2_/CdS and CuInS_2_/ZnS QDs).[[qv: 11e,18]] The cation exchange process in the synthesis of QDs usually leads to the formation of core/shell structures.^19^ Unlike the pyramidal CISeS QDs, the four corners of pyramids are not observed in CdS#3 sample (Figure 1b and Figure S3, Supporting Information). We assume that these CdS#3 QDs possess a possibly quasi‐octahedral shape that is formed by etching away the four corners of CISeS pyramids during the cation exchange process.[[qv: 11e,18]]

The HRTEM image of CdS#3 QDs (inset image in Figure 1b) shows a lattice spacing of 0.343 nm, which lies between 0.335 nm (indexed to the (111) plane of ZB phase CdS) and 0.351 nm (indexed to the (111) plane of ZB phase CdSe) that is well indexed to the (111) plane of the alloyed ZB phase CdSeS. The diffraction peaks of CdS#3 QDs in the XRD pattern (Figure 1e) are found in between the diffraction peaks of pure ZB phase CdS and CdSe, indicating that CdSeS crystallizes in an alloyed ZB phase, which is consistent with its SAED patterns (Figure S4a, Supporting Information). There are no diffraction peaks of CISeS core in the XRD patterns of CdS#3 QDs, which is attributed to the simultaneously decreasing size of the CISeS core and the increasing CdSeS shell thickness during the cation exchange process. As a result, the signal of the XRD pattern is dominated by the CdSeS shell materials in CdS#3 QDs and the contribution from the CISeS core is below the detection limit of XRD. These results show that the CdS#3 QDs are CISeS/CdSeS core/shell QDs with ZB phase CdSeS shell. In particular, there was a core‐etching effect at an early growth stage of g‐QDs:[[qv: 11e,18]] the pyramidal‐shaped CISeS core QDs were etched during the cation exchange process, resulting in CISeS/CdSeS core/shell QDs with the quasi‐octahedral shape.

Figure 1c displays TEM images of CdS#6 (with 6 mL of injected Cd/S precursors) QDs with an average size of 7.4 ± 0.6 nm and pyramidal shape. When increasing the injection volume of Cd/S precursors, the CdS#3 QDs with quasi‐octahedral shape was observed to grow at a faster rate at the four corners of the pyramids, leading to the larger size and restored pyramidal shape of CdS#6 QDs. This conclusion can also be drawn from the TEM images (Figure S5c,d, Supporting Information) of QDs at growth stages between CdS#3 and CdS#6 QDs.

The HRTEM image (inset image in Figure 1c), XRD (Figure 1e), and SAED (Figure S4b, Supporting Information) patterns of CdS#6 QDs demonstrated that their shell materials consisted of alloyed ZB phase CdSeS. Nonetheless, the lattice spacing and diffraction peaks are very close to those of the pure ZB phase CdS, indicating a higher S/Se ratio in the CdS#6 QDs as compared with CdS#3 QDs. The higher S/Se ratio in CdS#6 QDs is attributed to the fact that the composition of Se is constant (from CISeS QDs) during the entire reaction while the injection of S precursor leads to a decreasing ratio of Se and S in the resulting g‐QDs. TEM images of CdS#9 (with 20 mL of injected Cd/S precursors) QDs are shown in Figure 1d, exhibiting heterostructured g‐QDs with pyramidal shape and average size of 12.7 ± 0.9 nm. The HRTEM image (inset image in Figure 1d), XRD (Figure 1e), and SAED (Figure S4c, Supporting Information) patterns are well indexed to the ZB phase CdS, demonstrating the formation of the outer CdS shell in the subsequent growth stages of CdS#9 CdS. Moreover, the HRTEM image of CdS#9 g‐QDs (Figure S6, Supporting Information) clearly exhibits two (111) facets of the CdS shell, and the as‐measured angle (observed from [110] direction) of the projected triangle is 70.5°. This value is consistent with the 3D geometry of the pyramids, demonstrating that the CdS#9 QDs possess a pyramidal shape.

TEM images and the corresponding size distribution of other growth stages of g‐QDs are shown in Figures S5 and S7 (Supporting Information). Based on these results, we briefly summarize the growth dynamics of this type of heterostructured g‐QDs, as illustrated in Figure 1f: the initial CISeS core QDs with pyramidal shape were first etched so as to decrease the size and obtain a quasi‐octahedral shape. This is caused by an early cation exchange process (i.e., a common phenomenon in the growth of core/shell CuInSe(S)/Cd(Zn)S QDs[[qv: 11e,18]]), which leads to the formation of CISeS/CdSeS core/shell QDs with ZB phase CdSeS. The subsequent growth of the shell results in CISeS/CdSeS core/shell g‐QDs with higher S/Se ratio and increasing size of QDs, and the morphology of the QDs is restored to a pyramidal shape. With continued growth of QDs, an outer CdS shell with ZB phase is then formed on CISeS/CdSeS core/shell g‐QDs and leads to the growth completion of heterostructured pyramidal‐shaped CISeS/CdSeS/CdS g‐QDs.

The pyramidal heterostructured CISeS/CdSeS/CdS g‐QDs were successfully synthesized by using pyramidal‐shaped CISeS QDs as initial core materials. The crystal structure of their shell materials could be easily controlled and was demonstrated to be ZB phase CdSeS and CdS. Usually, the CdS shell has a WZ phase or mixed WZ and ZB phase, due to the high reaction temperature (240–300 °C).[[qv: 8b,11a,20]] In our case, due to the alloyed interfacial CdSeS layer that has the ZB crystalline structure, the subsequent growth of CdS (at 215 °C) conveniently crystallizes in the ZB phase. Generally, g‐QDs such as CdSe/CdS and PbSe/CdSe/CdSe with ZB phase shell materials possess less structural defects as compared to their WZ counterparts, leading to superior optical properties such as high PL QY and suppressed photoblinking,^21^ which are promising candidates for optoelectronic devices, for instance, high‐performance QDs‐based LEDs.^22^

### 2.2 Optical Properties of Heterostructured CISeS/CdSeS/CdS g‐QDs

**Figure 2:**
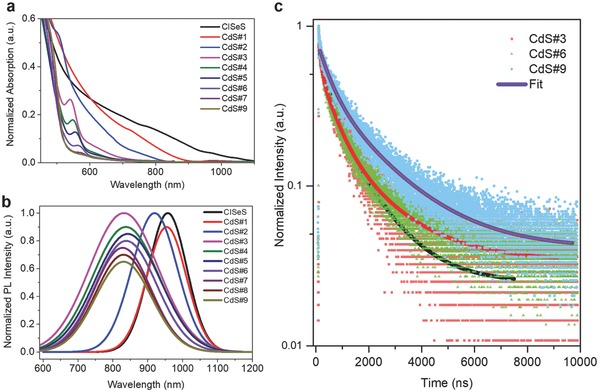
a) UV–vis absorption and b) PL spectra of heterostructured CISeS/CdSeS/CdS g‐QDs at different growth stages in toluene. c) PL lifetime of CdS#3, CdS#6, and CdS#9 g‐QDs in toluene.

**Figure** **2**a–c display the optical properties of heterostructured CISeS/CdSeS/CdS g‐QDs dispersed in solution. Figure 2a shows the absorption spectra of QDs at various growth stages. The initial CISeS QDs exhibit a typical absorption spectrum covering ultraviolet (UV)–visible–NIR region without apparent excitonic peaks.^15^ In contrast, with subsequent growth of the shell, the core/shell QDs exhibit a strong absorption feature in the UV–visible region, which is consistent with the typical absorption spectra of shell materials (i.e., CdSeS or CdS).^23^

There is a quantum‐confined feature in the absorption spectra of the QDs, which is expressed by the absorption peaks from absorption spectra of CdS#3 to CdS#6 QDs. The absorption peak positions of QDs are listed in Table S3 (Supporting Information). We attribute these absorption peaks to the CISeS/CdSeS core/shell QDs as there is a redshift and broadening of the peaks from CdS#3 (≈541 nm) to CdS#6 (≈567 nm) QDs with the gradual growth of the shell, and the peaks finally diminish in CdS#9 g‐QDs. All these results are consistent with the quantum‐confined effect of increasing sizes of QDs. The redshift and broadening of the absorption peaks is also consistent with the formation of CISeS/alloyed CdSeS core/shell QDs, as confirmed by the XRD (Figure 1e) and SAED patterns (Figure S4a,b, Supporting Information) of CdS#3 and CdS#6 QDs. The absorption spectrum of the final CdS#9 QDs exhibits no obvious absorption peak, since their absorption is dominated by the thick CdS shell.

PL spectra of CISeS/CdSeS/CdS g‐QDs at different growth stages in solution are also shown in Figure 2b and the detailed PL peak positions are listed in Table S4 (Supporting Information). All the QDs exhibit PL emission in the NIR region (over 700 nm), indicating that the origin of PL emission in these QDs originates from the core materials of CISeS, which exhibits typical emission in the 800–1000 nm range.^15^ We observe a continuous blueshift of PL peaks from CISeS to CdS#3 QDs, which is attributed to the decreasing size of the QDs caused by the core‐etching effect in the early growth stages.[[qv: 11e,18]] This is also consistent with the decreasing size from CISeS QDs to CdS#3 QDs observed in TEM images (Figure 1a,b and Figure S5, Supporting Information). In contrast, a redshift of PL peaks is displayed in the PL spectra of CdS#3 to CdS#5 (with 4 mL of injected Cd/S precursors). We infer that there is electron delocalization corresponding to the increasing thickness of CdSeS shell.[[qv: 7b,8b]] After growing the outer thick CdS shell, the PL peaks of CdS#7 (with 10 mL of injected Cd/S precursors) to CdS#9 QDs further blueshifted to ≈830 nm that is identical to the CdS#3 QDs. We attribute this blueshift to the outmost CdS shell that possesses wider bandgap than alloyed CdSeS. The wider bandgap is favorable to confine the electrons and results in less possibility of electrons delocalization. We further estimated the bandgap of all the QDs based on the Tauc plot (Figure S8, Supporting Information) of the QD's absorption spectra, and then used the bandgap and PL peaks to determine the Stokes shift of the QDs, as listed in Table S5 (Supporting Information). With the growth of the shell, the Stokes shift gradually increases from ≈173 nm (CISeS QDs) to ≈226 nm (CdS#7 QDs) and then remains constant for CdS#8 and CdS#9 QDs. The enhanced Stokes shift of g‐QDs with the increase of shell thickness is likely due to the strong delocalization of electrons into the shell region, which is consistent with other g‐QDs systems.[[qv: 12c,24]]

The PLQY of QDs was measured and shown in Figure S9a and Table S6 (Supporting Information). The PLQY of core/shell QDs is much higher than that of the initial CISeS QDs, which have a very low PLQY of ≈0.1%. For the PLQY of core/shell QDs, there is an increasing trend from CdS#1 (with 0.5 mL of injected Cd/S precursors) to CdS#6 QDs (maximal PLQY of ≈17%), and further growth of thicker shells leads to the decrease of PLQY from CdS#7 to CdS#9 QDs. The increasing PLQY with growth stages from CdS#1 to CdS#6 QDs is ascribed to the effective surface defects/traps passivation by inorganic shell growth, and the maximum PLQY of ≈17% was obtained from CdS#6 QDs, since most of the nonradiative recombination sites were passivated at this optimized growth stage.[[qv: 8b]] Generally, the PLQY is determined by both the radiative decay rate and nonradiative decay rate.^25^ From CdS#1 to CdS#6 QDs, the radiative decay rate decreases due to the electron delocalization and may cause the PLQY to decrease. Simultaneously, the formation of the inorganic shell can effectively passivate the surface defects/traps of QDs, leading to largely reduced number of surface recombination centers. This surface passivation results in lower nonradiative decay rate, which is the dominant process from CdS#1 to CdS#6 QDs and leads to enhanced PLQY. This maximal PLQY is also comparable to that of the recently reported PbSe/CdSe/CdSe g‐QDs (≈18%).[[qv: 21a]] For other CISeS QDs, they generally show PLQY around 5–10%,^18^, ^26^ which is less than our champion samples (17%). On the other hand, in the g‐QDs system, the formation of a very thick shell can create defects/dislocations, etc., at the interface or within the shell, which act as recombination centers and thus lead to the decreasing PLQY.[[qv: 8b,27]] In our case, as the thickness of the CdS shell increases, the strain due to the lattice mismatch between CdSeS and CdS could result in the formation of defects/dislocations at the interface of CdSeS/CdS shell or within the CdS shell, which could serve as nonradiative recombination centers, causing the reduced PLQY.[[qv: 8b,27]]

Figure 2c displays the transient PL decay of CdS#3, CdS#6, and CdS#9 g‐QDs, a triexponential decay is used to fit these curves, showing fitted average lifetimes of 1.28, 1.69, and 1.94 µs, respectively. Compared to CISeS core QDs with fitted average lifetime of ≈0.165 µs, there is an obvious prolonged lifetime for heterostructured CISeS/CdSeS/CdS g‐QDs with increasing shell thickness (PL lifetime of CdS#2, CdS#4, CdS#5, and CdS#8 QDs are shown in Figure S10, Supporting Information), as summarized in Table S7 (Supporting Information). The prolonged lifetime with increasing shell thickness of as‐synthesized g‐QDs is consistent with previous work on g‐QDs systems, which attributed this phenomenon to the delocalization of electrons in the shell region, while the holes are still confined in the core region.[[qv: 7b,8b]] In our case, we also suggested that the hole is still confined to the CISeS core while the electron is delocalized over the entire shell region, leading to a largely reduced electron–hole overlap and prolonged lifetime. The large difference (more than one order of magnitude) of average lifetime before and after shell growth is attributed to the designed pyramidal geometry for more efficient spatial separation of electrons and holes in as‐synthesized g‐QDs than conventional spherical‐shaped g‐QDs.[[qv: 4a,28]] As the lifetime of g‐QDs can be directly correlated with their electron/hole wave functions, we compared our pyramidal‐shaped g‐QDs with other spherical giant core/shell QDs, as summarized in Table S8 (Supporting Information). As‐synthesized pyramidal‐shaped CISeS/CdSeS/CdS g‐QDs exhibit a very long lifetime of ≈2 µs. This value is much higher than the corresponding value found for spherical CdSe/CdS g‐QDs (PL lifetime ≈40 ns), spherical CuInSe_2_/CuInS_2_ g‐QDs (PL lifetime ≈300 ns), and spherical PbS/CdS g‐QDs (PL lifetime ≈1 µs).[[qv: 1h,11c,12a]] All these results demonstrate the pyramidal g‐QDs may have particular optical properties compared to spherical g‐QDs.

### 2.3 Theoretical Modeling of Heterostructured CISeS/CdSeS/CdS g‐QDs

**Figure 3:**
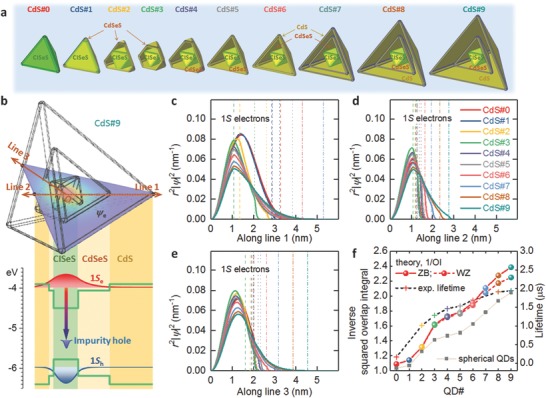
Theoretical modeling of the CISeS/CdSeS/CdS g‐QDs. a) Geometrical models of the series of g‐QDs (CdS#0‐9). Each edge of each component of the QDs is rounded by a radius of 0.3 nm. b) Electronic band structure with energy levels and wave functions of 1S electrons, impurity holes and 1S holes in a g‐QD (CdS#9). c–e) Normalized radial distribution function of 1S electrons in the series of g‐QDs along Line 1, Line 2, and Line 3, respectively. The Lines 1–3 are vectors pointing from the origin to the vertex, face center, and edge center of the tetrahedron QD, respectively, as demonstrated in (b). The vertical lines show the positions of the surfaces of CISeS (dashed), CdSeS (dotted), and CdS (dashed‐dotted) of each g‐QD. f) Inverse squared overlap integral, 1/OI, of the 1S electrons and impurity holes in the series of g‐QDs with two different crystal structures, ZB and WZ, for the CdSeS shell and CdS shell. The inverse squared overlap of pyramidal QDs is much higher than that of the spherical QDs (ZB). The experimental lifetime is plotted for comparison (right axis).

We calculated the wave functions of the electrons in the g‐QDs to gain a better understanding of the electron behavior and to describe the prolonged PL lifetime with increasing shell thickness. We estimated the shape and size of each component in the g‐QDs at different growth stages according to the growth dynamics of the series of g‐QDs. In our models (**Figure** **3**a), we further assume that (1) the etching process of the CISeS core size stopped when the injection volume of the mixed Cd and S precursors exceeded 2.5 mL (as for the cases of CdS#3 to 9 QDs), (2) the growth process of the CdSeS shell stopped when the injection volume became greater than 4 mL, and (3) the outermost CdS shell was grown only if the injection volume was greater than 4 mL (as for the cases of CdS#5 to 9 QDs). The wave functions of the 1S electrons were calculated by solving the stationary Schrödinger equation with the bulk band alignment (Figure 3b and Figure S11, Supporting Information, for more details). The details of the geometrical and physical parameters are listed in Tables S9 and S10 (Supporting Information), respectively.

Our calculations show that the eigenenergies of the 1S electrons exceed the CdSeS energy barrier for the CdS#1‐9 QDs. Thus, the electrons have a higher probability of being found in the shell layers than the holes. The radial probability distributions of the 1S electrons along three different lines are shown in Figure 3c–e. The delocalization effect of the 1S electrons becomes prominent for QDs with thick CdS shell layers, for example, CdS#6 to 9 QDs. Moreover, the wave functions of 1*S* electrons in the g‐QDs are spatially anisotropic because of the non‐spherical shapes of the QDs, yielding the direction‐dependent localization degree of the 1*S* electrons (Figure S12a, Supporting Information). Unlike isotropic spherical QDs, the electron wave functions spread considerably out of the CISeS core along Line 1 (from the origin to the vertex of the tetrahedron‐shaped QDs), whereas the electrons are better confined in the direction of Line 2 (from the origin to the face center of the QDs). Along Line 3 (from the origin to the edge center), the electron wave functions have slightly better confinement than those along Line 1. In contrast, the holes wave functions are almost confined in the core region (Figure S13, Supporting Information), indicating the quasi‐type II band structure of as‐synthesized g‐QDs. As a consequence of the geometrical anisotropy and asymmetric electron–hole distribution, Line 2 is the most efficient path for electron tunneling from the CISeS core to the QD surface (Figure S12b, Supporting Information). The electrons tunneling to the QD surface can be used in many optoelectronic applications such as PEC and QDSCs,[[qv: 11c,12a,29]] where the tunneling rate depends on both the probability density of the electrons at the QD's surface and the lifetime of the photoexcited electrons.

To qualitatively evaluate the lifetime of the photoexcited carriers in the g‐QDs, we calculated the squared overlap integral (OI) of the photoexcited carriers in the series of g‐QDs, CISeS to CdS#9 QDs. Upon photoexcitation, the 1S holes nonradiatively moved from the valence band to the impurity in the core.^30^ The luminescence of the g‐QDs is attributed to the radiative recombination of the conduction band 1S electrons with the impurity holes.^30^ The squared OI between 1S electrons and impurity holes can be written as

(1) $$\mathrm{OI}=N_{\mathrm{impurity}}\frac{1}{V_{\mathrm{core}}}\int_{\mathrm{core}}{\mathrm{OI}}_{0}(r_{\mathrm{impurity}})dV_{\mathrm{impurity}}$$

with

(2) $${\mathrm{OI}}_{0}(r_{\mathrm{impurity}})=\frac{|\int \psi_{\mathrm{electron}}(r)\psi_{\mathrm{hole}}(r;r_{\mathrm{impurity}})dV{|}^{2}}{\int |\psi_{\mathrm{electron}}(r){|}^{2}dV\int |\psi_{\mathrm{hole}}(r;r_{\mathrm{impurity}}){|}^{2}dV}$$

Here, ψ_electron_ and ψ_hole_ are wave functions of the 1S electron state and impurity hole state, respectively, and *N*_impurity_ is the number of impurities in the CISeS core (see the Supporting Information for description of impurity holes). The PL lifetime should be proportional to the inverse squared overlap integral, 1/OI. Figure 3f shows that the inverse squared overlap integral continuously increases from core CISeS QDs to core/shell CdS#9 g‐QDs. This trend qualitatively agrees with the measured lifetime, indicating that the prolonged lifetime in the experiments resulted from the reduced overlap between 1S electrons and impurity holes. To show the beneficial role of the geometrical anisotropy, we compare the lifetime of pyramidal QDs with that of spherical QDs. The gray square dots in Figure 3f show the calculated lifetime of spherical QDs. The spherical QDs have similar configurations of the shells to the corresponding pyramidal QDs (see Table S11, Supporting Information, for geometrical parameters). The length of the most efficient path of electron tunneling for each spherical QD is set as the same as the corresponding pyramidal QD. As shown, the inverse square overlap integral of the pyramidal QDs is greater than that of the spherical QDs for the two series of QDs, indicating that anisotropic shapes of QDs can prolong the lifetime of the electrons in them. Moreover, we show that the g‐QDs with the ZB crystal structure of CdSeS and CdS have longer lifetime than those with WZ crystal structure of the shell layers, confirming that the ZB phase of CdSeS and CdS in our experiments played a beneficial role in creating the good optical properties (long PL lifetime with high PLQY). These simulation results indicate the quasi‐type II band structure of these g‐QDs, which is consistent with the experimental data.

### 2.4 Optoelectronic Devices Based on Heterostructured CISeS/CdSeS/CdS g‐QDs

We used heterostructured pyramidal‐shaped CISeS/CdSeS/CdS g‐QDs to fabricate a photoanode (detailed fabrication process is shown in the Supporting Information) for PEC hydrogen production. Before fabricating QDs‐based optoelectronic devices, we further optimized the shell thickness of an interfacial CdSeS layer of CdS#6 g‐QDs (the optimized synthesis process is described in the Supporting Information and the optical characterization is shown in Figure S14, Supporting Information). To fabricate QDs‐sensitized photoanodes, the as‐synthesized CdS#6 and CdS#9 g‐QDs were deposited into the mesoporous TiO_2_ films via electrophoretic deposition (EPD). A TEM image and relevant energy‐dispersive X‐ray spectroscopy (EDS) spectra of TiO_2_/CdS#6 g‐QDs/ZnS heterostructure are shown in Figure S15 (Supporting Information), indicating that the g‐QDs (denoted by white dashed circles) are uniformly dispersed in the TiO_2_ films.

To further verify this conclusion, cross‐sectional scanning electron microscopy (SEM) imaging and relevant EDS measurements (Figure S16, Supporting Information) of the CdS#6 g‐QDs‐sensitized photoelectrode were carried out. The mesoporous TiO_2_ films show an approximate thickness of ≈20.1 µm (Figure S16a, Supporting Information). The relative mass concentration of CdS#6 g‐QDs/TiO_2_ heterostructure is reported in Figure S16b (Supporting Information), confirming the existence of the main chemical composition (Cd, Se, S, Si, Ti, and O) in the CdS#6 g‐QDs‐sensitized photoanode. The element of Cu and In in CISeS core QDs are not detected due to the relatively large volume of CdSeS/CdS shell materials in these g‐QDs, while the elements of Cd, S, and Se are main elements in the g‐QDs and show very homogeneous distribution in the 2D EDS mapping (Figure S16c–e, Supporting Information) imaging.

**Figure 4:**
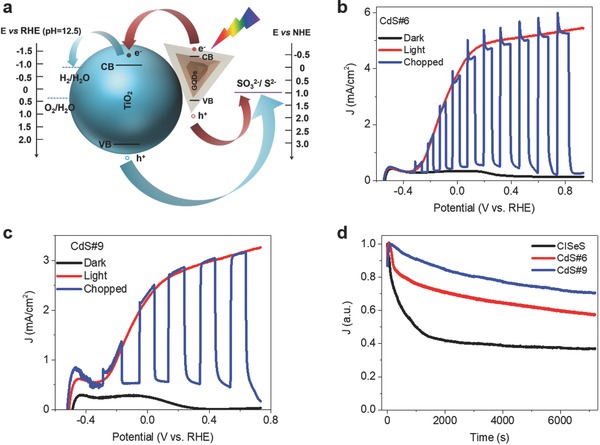
a) Scheme and predictable band alignment and of heterostructured CISeS/CdSeS/CdS g‐QDs‐based photoelectrodes. Linear sweep voltammetry of b) TiO_2_/CdS#6 g‐QDs/ZnS and c) TiO_2_/CdS#9 g‐QDs/ZnS systems in the dark and under AM 1.5 G irradiation at 100 mW cm^−2^. d) Normalized steady‐state current density–time (*J*–*t*) curves of CISeS QDs (black curve), CdS#6 g‐QDs and CdS#9 g‐QDs‐decorated photoanodes at 0.6 V versus RHE under standard 1 sun illumination.

**Figure** **4**a shows the scheme and predictable band alignment of pyramidal‐shaped CISeS/CdS/CdS g‐QDs‐sensitized photoanode. The CISeS/CdS/CdS g‐QDs form a staggered band alignment with TiO_2_ that allows for efficient charge separation, in which situation the photogenerated electrons are injected into TiO_2_ and move to the counter electrode (Pt) to conduct water reduction and enable hydrogen generation.^31^ The hole scavengers (i.e., Na_2_S and Na_2_SO_3_) in the electrolyte are consumed by photogenerated holes. A typical three‐electrode electrochemical cell was employed to estimate the PEC performance of these g‐QDs‐sensitized photoanodes. Before PEC measurements, an extra inorganic ZnS capping layer is deposited on the QDs‐sensitized photoanode by successive ionic layer adsorption and reaction (SILAR) method to avoid photocorrosion of the electrodes. All of the PEC measurements are conducted under standard 1 sun illumination (AM 1.5 G, 100 mW cm^−2^).

As shown in Figure 4b,c, the CdS#6 and CdS#9 g‐QDs‐sensitized photoanodes yield a saturated photocurrent density of ≈5.5 and ≈3 mA cm^−2^ at ≈0.6 V versus the reversible hydrogen electrode (RHE). The performance of our g‐QDs‐based PEC cells is comparable to PbS/CdS g‐QDs‐based PEC system.[[qv: 11c]] In contrast, the bare CISeS QDs‐sensitized photoanode shows a lower saturated photocurrent density of ≈2.1 mA cm^−2^ (Figure S17, Supporting Information). To eliminate the possible photoresponse from TiO_2_ photoelectrode, the control sample of blank TiO_2_ photoelectrode only exhibits a very low saturated photocurrent density of ≈0.25 mA cm^−2^ (Figure S18, Supporting Information). Although the CdS#6 and CdS#9 g‐QDs possess less light absorption in visible–NIR region than bare CISeS QDs, the prolonged lifetime for efficient electron–hole separation and largely enhanced PL QY for suppressed surface charge carrier recombination are very favorable in general PEC systems,^32^ leading to higher saturated photocurrent density in CdS#6 and CdS#9 g‐QDs‐sensitized photoanodes than the CISeS QDs‐sensitized photoanode.

Steady‐state current density–time (*J*–*t*) curves of CISeS QDs (black curve), CdS#6 g‐QDs, and CdS#9 g‐QDs‐modified photoanodes measured at 0.6 V versus RHE are exhibited in Figure 4d. The curves are normalized by dividing the maximum photocurrent density (decay from the value of 1), allowing us to visualize the decay trend. The photocurrent density of TiO_2_/bare CISeS QDs/ZnS‐based PEC cell exhibits a rapid decay of photocurrent density, maintaining only 40% of its initial value after 2 h illumination. In contrast, the CdS#6 and CdS#9 g‐QDs‐based PEC cells present a lower percentage decay, maintaining ≈60 and ≈70% of its initial value after 2 h illumination. This enhanced stability of g‐QDs‐based PEC cells is ascribed to the construction of CdSeS/CdS thick shell on the CISeS core QDs for improved photo‐ and chemical stability, which is comparable with the best reported CdS QDs‐based PEC systems,^14^ demonstrating the long‐term stability of these pyramidal g‐QDs‐based PEC system.

In addition, CdS#6 g‐QDs were used as light harvesters to fabricate QDSCs. As shown in Figure S19 (Supporting Information), the preliminary device based on CdS#6 g‐QDs shows promising performance (PCE = 1.5%, *J*_sc_ = 5 mA cm^−2^, *V*_oc_ = 0.527 V, and FF = 57%) under 1 sun simulated sunlight (AM 1.5G, 100 mW cm^−2^), suggesting the versatility of CISeS/CdSeS/CdS g‐QDs for applications in optoelectronic devices.

## 3 Conclusions and Perspectives

In summary, we synthesized heterostructured CISeS/CdSeS/CdS g‐QDs with pyramidal shape and NIR emission via using a facile two‐step approach. The morphology and crystal structure characterizations demonstrated the growth dynamics of as‐synthesized heterostructured g‐QDs with shell materials of ZB phase CdSeS and CdS. The shell thickness of as‐synthesized g‐QDs can be tuned by varying the injection volume of precursors. The as‐obtained g‐QDs have high PLQY, long lifetime, and NIR‐active absorption and emission spectra. In addition, the prolonged PL lifetime with increasing shell thickness indicates the reduced spatial electron–hole overlap benefiting from core/shell/shell pyramidal structure and their quasi‐type II band structure. This conclusion of quasi‐type II band structure in these g‐QDs is consistent with simulation results, showing their potential applications in QDs‐based optoelectronic devices. The PEC cells and QDSCs based on heterostructured CISeS/CdSeS/CdS g‐QDs exhibit excellent optoelectronic performance in terms of efficient charge carrier separation and transfer in such pyramidal‐shaped g‐QDs.

This synthetic method may also be used for other nonspherical g‐QDs systems, such as pyramid giant CuInSe_2_/CuInSe*_x_*S_1−_ *_x_*/CuInS_2_, SnSe/SnSe*_x_*S_1−_ *_x_*/SnS with well‐controlled crystal structure and well‐separated electron–hole wave function. In addition, future optimizations should concentrate on engineering the shell structure (for instance, CdSe or CuInS for enhanced light absorption) of these pyramidal g‐QDs. Overall, these results indicate that heterostructured pyramidal‐shaped g‐QDs can efficiently control the electron–hole wave function of g‐QDs and are promising for the fabrication of efficient and stable optoelectronic devices.

## 4 Experimental Section

*Synthesis of “Giant” CISeS/CdSeS/CdS QDs*: CISeS QDs were first synthesized by the method described elsewhere.^15^ Typically, 1 mmol of copper(I) iodide (Sigma‐Aldrich), 1 mmol of indium(III) acetate (Sigma‐Aldrich), 1 mL of oleylamine (OLA) (technical grade, 70%, Sigma‐Aldrich), and 5 mL of 1‐dodecanethiol (DDT) (Sigma‐Aldrich) were loaded in a three‐necked flask (50 mL). The reaction mixture was degassed under vacuum at 90 °C for 30 min and then heated to 140 °C for ≈15 min. The OLA/DDT‐Se solution (2 m) was prepared by mixing 2 mmol of Se powder (Sigma‐Aldrich) with 0.5 mL of DDT and 1.5 mL of OLA. The temperature of flask was then raised to 210 °C and OLA/DDT‐Se solution (2 m) was injected into the reaction mixture. The reaction solution was then maintained at 210 °C for 10 min and heated to 235 °C for 15 min to grow the QDs. As‐synthesized CISeS QDs were precipitated with ethanol, centrifuged, and redispersed in toluene.

“Giant” CISeS/CdSeS/CdS core/shell QDs were synthesized via the modified approaches reported by Klimov's group.[[qv: 11e,18]] For growth of the shell on CISeS QDs, 1 mL of 0.2 m Cd‐oleate prepared by dissolving the CdO in OA and ODE was first injected into the reaction solution (≈1 × 10^−7^ mol of CISeS QDs in 5 mL of ODE) at 160 °C, then a mixture of 0.2 m Cd‐oleate (10 mL), S (2 mmol, Sigma‐Aldrich) powder dissolved in 2 mL of trioctylphosphine (TOP) (97%, Sigma‐Aldrich) and 8 mL of ODE was added dropwise into the reaction solution heated to 215 °C at the rate of 4 mL h^−1^ for 5 h. During the injection of Cd/S precursors (20 mL in total), the resulting QDs at different intermediate steps were taken out and precipitated with ethanol, centrifuged and redispersed in toluene for further characterization. Detailed injection volumes of Cd/S precursors at diverse intermediate steps and corresponding sample labels are listed in Table S1 (Supporting Information).

CdS#6 g‐QDs with optimized alloyed shell thickness were synthesized by introducing a CdSeS interfacial layer with additional Se precursor: 1 mL of 0.2 m Cd‐oleate was first injected into the reaction solution (≈1 × 10^−7^ mol of CISeS QDs in 5 mL of ODE and 5 mL of OLA) at 160 °C, then a mixture of 0.2 m Cd‐oleate (1.5 mL), 0.4 m Se in TOP (0.75 mL), and 0.4 m S in ODE (0.75 mL) was added dropwise into the reaction solution heated to 215 °C at the rate of 4 mL h^−1^, followed by injection of a mixture of 0.2 m Cd‐oleate (1.5 mL) and 0.2 m S in ODE (1.5 mL) under 215 °C at the rate of 4 mL h^−1^.

*Fabrication of QDs‐Sensitized Photoelectrode and Solar Cells*: Colloidal QDs in toluene were deposited into double‐layer TiO_2_ mesoporous films (prepared by doctor‐blading technique^13^) by using EPD approach.^33^ An applied bias of 50 V was added on the two electrodes for 30 min. Subsequently, the as‐deposited QDs‐sensitized electrodes were rinsed with toluene to remove the unbound QDs on the surface of the TiO_2_ film. The positions of two QDs‐sensitized electrodes were then exchanged and an applied bias of 75 V was added for another 90 min. These electrodes were then dipped into a hexadecyltrimethylammonium bromide (CTAB) solution (10 mg mL^−1^ in methanol) for 1 min and rinsed with methanol for 1 min; these procedures were repeated twice. Next, two layers of ZnS were deposited on QDs‐sensitized photoanodes by using the SILAR method so as to avoid photocorrosion. Finally, an insulating glue was employed to cover the photoanode's surface excluding the active area (with size of ≈0.15 cm^2^) to finalize device fabrication.

For fabrication of QDSCs, the as‐prepared anode was further coated with silica (the anode was immersed in 0.01 m tetraethylorthosilicate/ethanolic solution for 2 h at 35 °C). The electrolyte was prepared by mixing polysulfide in H_2_O/methanol (1/1, v/v) (1 m Na_2_S, 1 m S, and 0.1 m NaOH). The Cu_2_S counter electrode was deposited by soaking the brass in hydrochloric acid (HCl, 30%) at 70 °C for 10 min. Then, as‐treated brasses were dipped in a polysulfide electrolyte (2 m S, 2 m Na_2_S, and 0.2 m NaOH) solution for 10 min to produce Cu_2_S. In the end, QDSCs were constructed by sandwiching the Cu_2_S counter electrode and the QDs‐modified photoanode via using a plastic spacer (thickness of ≈25 µm).

*Characterization*: The UV–visible absorption spectra were acquired by using a Cary 5000 UV–visible–NIR spectrophotometer (Varian). The fluorescence spectra and transient PL spectra of the QDs were obtained via a Fluorolog‐3 system (Horiba JobinYvon). TEM images and SAED patterns of QDs were measured using a JEOL 2100F TEM. XRD patterns were obtained by using a Panalytical X‐Pert PRO MRD with Cu Kα radiation. Cross‐sectional SEM images and EDS mapping of QDs‐sensitized photoanodes were characterized by employing JSM‐7401F SEM. The PEC performance of the QDs‐sensitized photoanodes was measured by using a three‐electrode electrochemical cell with a Pt counter electrode, a QDs/TiO_2_ working electrode, and a saturated Ag/AgCl reference electrode. Electrochemical workstation (CHI‐760D, with sweep rate of 20 mV s^−1^) was used to conduct linear sweep voltammetry measurements. Photocurrent density–voltage (*J–V*) curves were measured by using a (Sciencetech SLB‐300A) Compact Solar Simulator Class AAA with simulated 1 sun illumination (AM 1.5 G, 100 mW cm^−2^). A Si reference diode (Sciencetech) was employed to validate the standard 1 sun irradiation (100 mW cm^−2^) and calibrate the distance (≈30 cm) between sun simulator and photoanode before each measurement.

*Theoretical Method*: A commercial software of COMSOL was employed to solve the stationary Schrödinger equation for the 1S electrons and holes. The electron and hole potentials as a function of position were approximated as the lowest unoccupied molecular orbital and the highest occupied molecular orbital levels of their bulk materials. Figure S11 (Supporting Information) shows the band diagrams. The bulk values for the effective masses of electrons and holes were used. The physical parameters are summarized in Table S10 (Supporting Information). We neglect the interaction between electrons and holes for simulations. The wave functions were computed from the effective mass Schrödinger equation. The appropriate boundary conditions at the interfaces were used to solve this equation and the wave functions were normalized as below: ∫|ψ|^2^d*V* = 1. The wave function of an impurity hole state can be expressed by the equation

(3) $$\psi_{\mathrm{hole}}(r;r_{\mathrm{impurity}})=A\exp\left(-\frac{{\left(r-r_{\mathrm{impurity}}\right)}^{2}}{2L_{h}^{2}}\right)$$

In this equation, **r**_impurity_ represents the position of the impurity in the CISeS core; the coefficient *A* can be derived from the equation: ∫|ψ_hole_|^2^d*V* = 1; The scale of hole *L*_h_ is set to be 0.3 nm (*L*_h_ is generally much less than QD size). From the experimental data, the molar ratio of Cu:In in CISeS is 1:1.2. Based on this point, the molar mass and mass density of CISeS were employed to calculate the numbers of Cu atoms and In atoms. The number of In atoms that occupy the Cu sites is shown in Table S12 (Supporting Information).

## Conflict of Interest

The authors declare no conflict of interest.

## Supporting information

Supplementary
