# Supplementary material for: Optoelectronic Properties in Near‐Infrared Colloidal Heterostructured Pyramidal “Giant” Core/Shell Quantum Dots
Source: Adv Sci (Weinh). 2018 Jul 3;5(8):1800656. doi: 10.1002/advs.201800656 (PMC6097093; doi:10.1002/advs.201800656)
Supplement: Supplementary file 1 — Supplementary [file ADVS-5-1800656-s001.pdf]

Copyright WILEY-VCH Verlag GmbH & Co. KGaA, 69469 Weinheim, Germany, 2018.

ADVANCED SCIENCE
Open Access

Supporting Information

for *Adv. Sci.,* DOI: 10.1002/advs.201800656

Optoelectronic Properties in Near-Infrared Colloidal Heterostructured Pyramidal "Giant" Core/Shell Quantum Dots

Xin Tong, Xiang-Tian Kong, Chao Wang, Yufeng Zhou, Fabiola Navarro-Pardo, David Barba, Dongling Ma, Shuhui Sun, Alexander O. Govorov, Haiguang Zhao, Zhiming M. Wang,* and Federico Rosei**

Supporting Information

***Title** (Optoelectronic Properties in Near-infrared Colloidal Heterostructured Pyramidal "Giant" Core/shell Quantum Dots)*

*Xin Tong, Xiang-Tian Kong, Chao Wang, Yufeng Zhou, Fabiola Navarro-Pardo, David Barba, Dongling Ma, Shuhui Sun, Alexander O. Govorov, Haiguang Zhao *, Zhiming M. Wang *, and Federico Rosei **

Table S1. Detailed information for injection volumes of mixed Cd and S precursors and corresponding sample labels during shell growth of heterostructured CISeS/CdSeS/CdS g-QDs.

**Injection Volumes**	**0.5 mL**	**1.4 mL**	**2.5 mL**	**3.2 mL**	**4 mL**	**6 mL**	**10 mL**	**15 mL**	**20 mL**
**Sample Labels**	CdS#1	CdS#2	CdS#3	CdS#4	CdS#5	CdS#6	CdS#7	CdS#8	CdS#9

Table S2. Size distribution of bare CISeS QDs and corresponding heterostructured CISeS/CdSeS/CdS core/shell QDs (CdS#1 to CdS#9).

**Sample Labels**	**CISeS**	**CdS#1**	**CdS#2**	**CdS#3**	**CdS#4**	**CdS#5**
**Size (nm)**	5.5±0.7	5.4±0.6	4.7±0.5	4.0±0.3	5.2±0.5	6.4±0.5
**Sample Labels**	**CdS#6**	**CdS#7**	**CdS#8**	**CdS#9**	**CdS#6**	**CdS#7**
**Size (nm)**	7.4±0.6	8.6±0.6	10.6±0.7	12.7±0.9	7.4±0.6	8.6±0.6

Table S3. Absorption peak positions of heterostructured CISeS/CdSeS/CdS QDs (CdS#2 to CdS#7).

**Sample Labels**	**CdS#2**	**CdS#3**	**CdS#4**	**CdS#5**	**CdS#6**	**CdS#7**
**Absorption Peak (nm)**	506	541	548	556	568	576

Table S4. PL peak positions of CISeS core QDs and corresponding core/shell QDs (CdS#1 to CdS#9).

Sample Labels	CISeS	CdS#1	CdS#2	CdS#3	CdS#4	CdS#5	CdS#6	CdS#7	CdS#8	CdS#9
**PL Peak (nm)**	~957	~954	~919	~830	~837	~842	~838	~830	~830	~830

Table S5. Stokes shift of CISeS QDs to CdS#9 g-QDs.

Sample	E_g (eV)	Abs peak(nm)	PL peak(nm)	Stokes Shift (nm)
**CISeS**	1.58	784	957	173
**CdS#1**	1.60	774	954	180
**CdS#2**	1.70	729	919	190
**CdS#3**	1.95	635	830	195
**CdS#4**	1.95	635	837	202
**CdS#5**	1.95	635	842	207
**CdS#6**	2.00	620	838	218
**CdS#7**	2.05	604	830	226
**CdS#8**	2.05	604	830	226
**CdS#9**	2.05	604	830	226

Table S6. Quantum yield (QY) of bare CISeS QDs and relevant core/shell QDs (CdS#1 to CdS#9).

Sample Labels	CISeS	CdS#1	CdS#2	CdS#3	CdS#4	CdS#5	CdS#6	CdS#7	CdS#8	CdS#9
**QY (%)**	0.1	0.5	3.2	8	10.5	14.1	17	10.7	7.5	7.3

Table S7. Average lifetime of the CISeS core QDs and corresponding core/shell QDs (CdS#2 to CdS#6, CdS#8 and CdS#9).

Sample Labels	CISeS	CdS#2	CdS#3	CdS#4	CdS#5	CdS#6	CdS#8	CdS#9
**Lifetime($\tau_{measure}$)**	165±4 ns	1.01 μs	1.28 μs	1.46 μs	1.53 μs	1.69 μs	1.91 μs	1.94 μs

Table S8. PL lifetime of spherical g-QDs with respect to as-synthesized pyramidal-shaped g-QDs.

QDs type	PL lifetime (ns)	Reference
**Spherical CdSe/CdS**	~ 40	[12a]

**Spherical $CuInSe_2/CuInS_2$**	~ 300	[1h]
**Spherical PbS/CdS**	~ 1000	[11c]
**Pyramidal-shaped CISeS/CdSeS/CdS**	~ 2000	This work

Table S9. Geometrical parameters used in modeling.

**Sample name**	**CdS#0**	**CdS#1**	**CdS#2**	**CdS#3**	**CdS#4**	**CdS#5**	**CdS#6**	**CdS#7**	**CdS#8**	**CdS#9**
**Size (nm)**	5.5	5.4	4.7	4.0	5.2	6.4	7.2	8.8	10.7	12.7
**Overall shape**	Tetra		Octahedron					Tetrahedron		
**CISeS size (nm)**	5.5	4.9	3.0	2.5	2.5	2.5	2.5	2.5	2.5	2.5
**CdSeS size (nm)**	--	0.5	1.7	1.5	2.7	3.9	3.9	3.9	3.9	3.9
**CdS size (nm)**	--	--	--	--	--	0.0	0.8	2.4	4.3	6.3

Table S10. Physical parameters used in modeling.

	E_v **(eV)**	E_c **(eV)**	E_g **(eV)**	m_e / m_0	m_{hh} / m_0
**CISeS**	-5.78	-4.50	1.28	0.13	0.79
**CdSeS (ZB)**	-6.19	-4.06	2.13	0.17	0.57
**CdS (ZB)**	-6.4	-3.9	2.5	0.21	0.68
**CdSeS (WZ)**	-6.17	-4.03	2.14	0.17	0.57
**CdS (WZ)**	-6.37	-3.83	2.54	0.21	0.68
**CdSe**	-5.97	-4.22	1.75	0.13	0.45
**Vacuum**	-9.8	0	--	1	

Table S11. Geometry parameters of spherical QDs calculated in Figure 3f.

**CdS #**	**0**	**1**	**2**	**3**	**4**	**5**	**6**	**7**	**8**	**9**
**Core radius**	1.91	1.82	1.49	1.26	1.26	1.26	1.26	1.26	1.26	1.26
**CdSeS shell thickness**	-	0.03516	0.10547	0.21093	0.3164	0.38671	0.38671	0.38671	0.38671	0.38671
**CdS shell thickness**	-	-	-	-	-	-	0.17579	0.49219	0.87891	1.30079

Core radius = Effective radius of CuInSeS (nm); CdSeS shell thickness = Thickness along best tunneling path of CdSeS (nm); CdS shell thickness = Thickness along best tunneling path of CdS (nm).

Table S12. Estimated number of impurities in CISeS core.

	**CISeS**	**CdS#1**	**CdS#2**	**CdS #3-#9**
**Impurity species**	In_{Cu} (1/11 of Cu sites are occupied by In atoms)			

**Number of Cu atoms in CISeS core**	288	249	137	83
**Number of In atoms in CISeS core**	345	299	164	100
**Number of In_{Cu}**	29	25	13	8

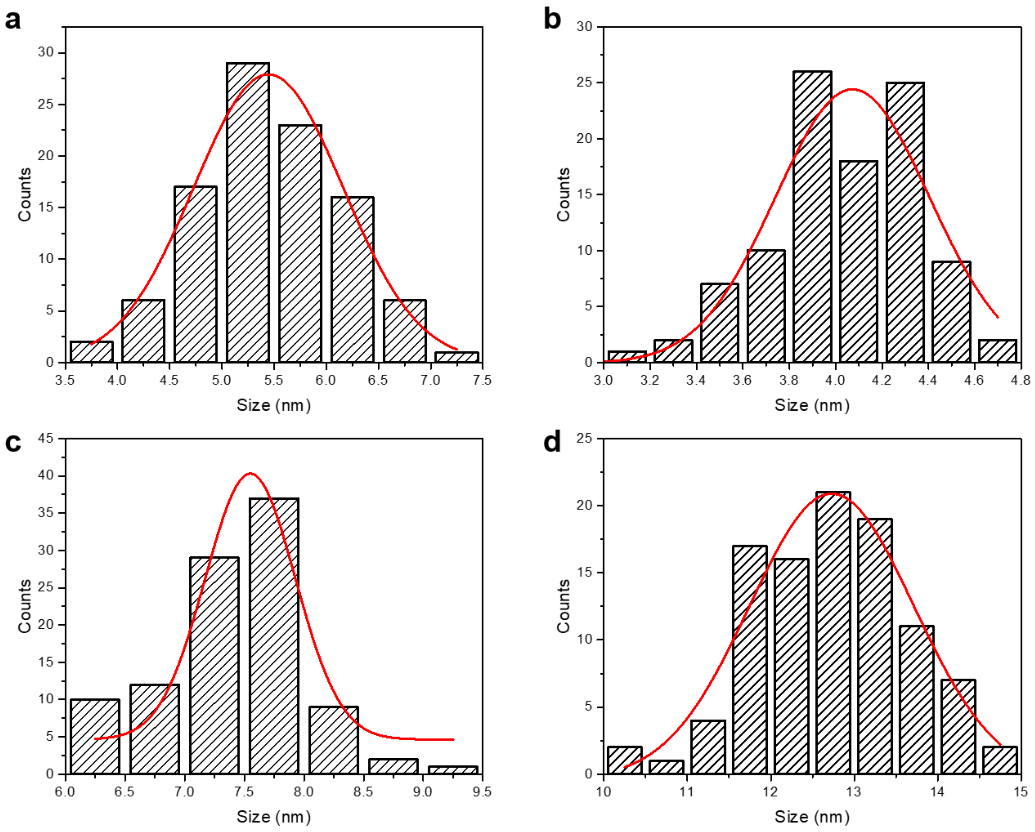

Figure S1. Size distribution of (a) CISeS, (b) CdS#3, (c) CdS#6 and (d) CdS#9 QDs, showing sizes of 5.5±0.7 nm, 4.0±0.3 nm, 7.4±0.6 nm and 12.7±0.9 nm, respectively. Sizes are measured as the height of projected triangles [15] (distance from a vertex to the middle of the opposite side) in TEM images for at least 100 QDs.

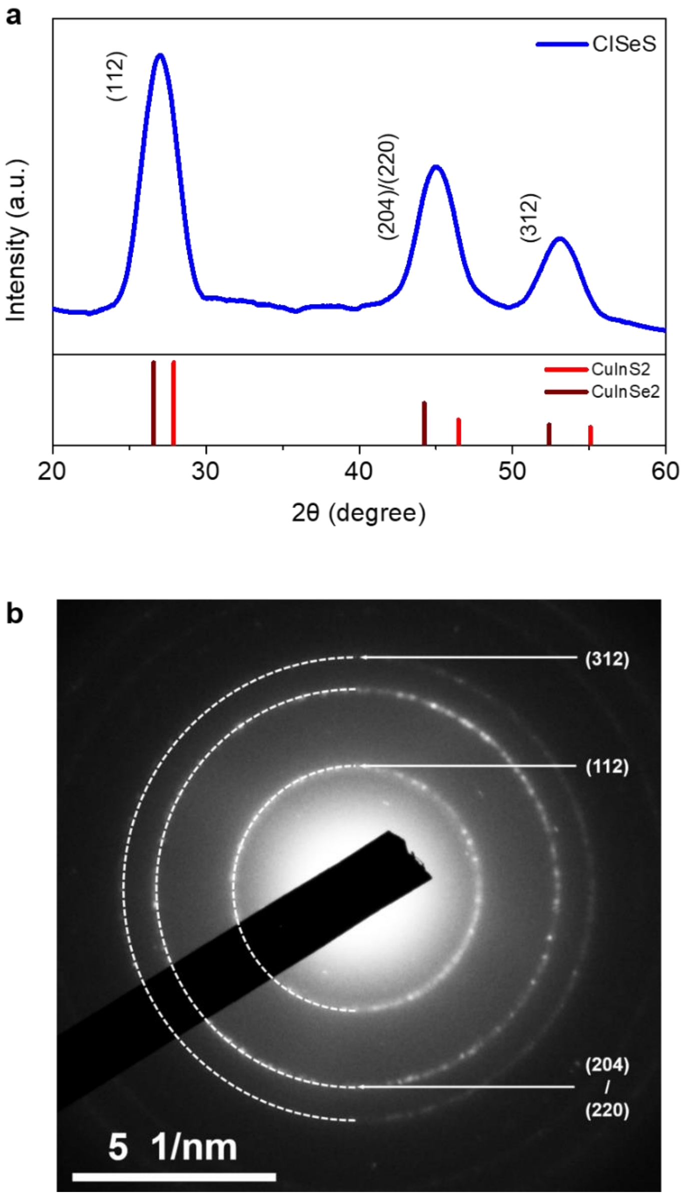

Figure S2. (a) XRD pattern of CISeS QDs films prepared by drying the dropped CISeS QDs solution on the top of silicon substrate. (b) SAED pattern of CISeS QDs. All of the diffraction peaks lie between (112), (204/220) and (312) facets of pure chalcopyrite $CuInS_2$ (JCPDS card no. 03-065-1572) and pure chalcopyrite $CuInSe_2$ (JCPDS card no. 00-040-1487), indicating the chalcopyrite phase and alloyed nature of as-prepared CISeS core QDs.

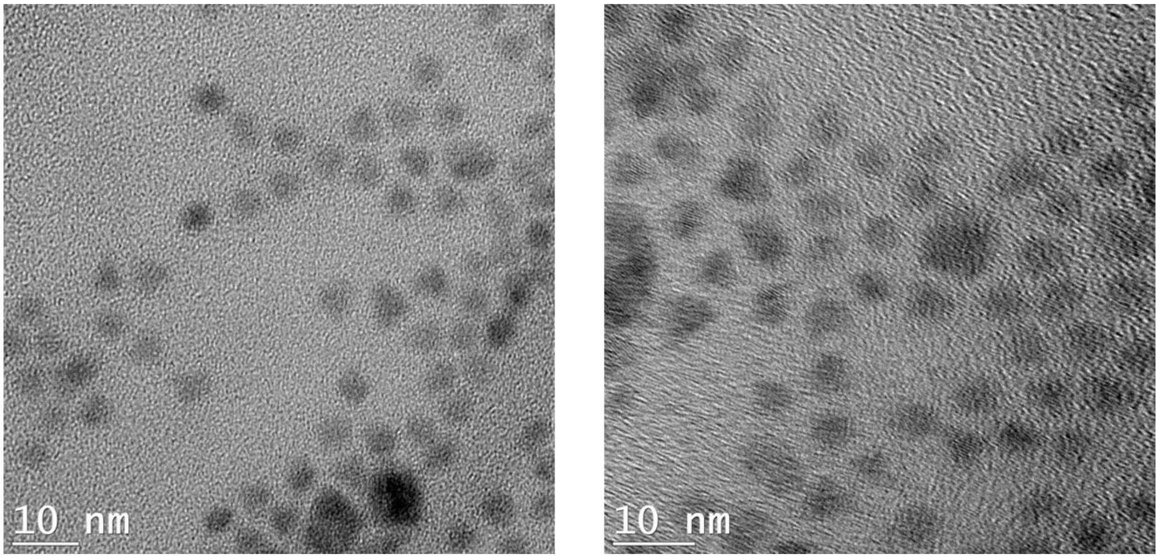

Figure S3. TEM of CdS#3 QDs, showing possible quasi-octahedral shape.

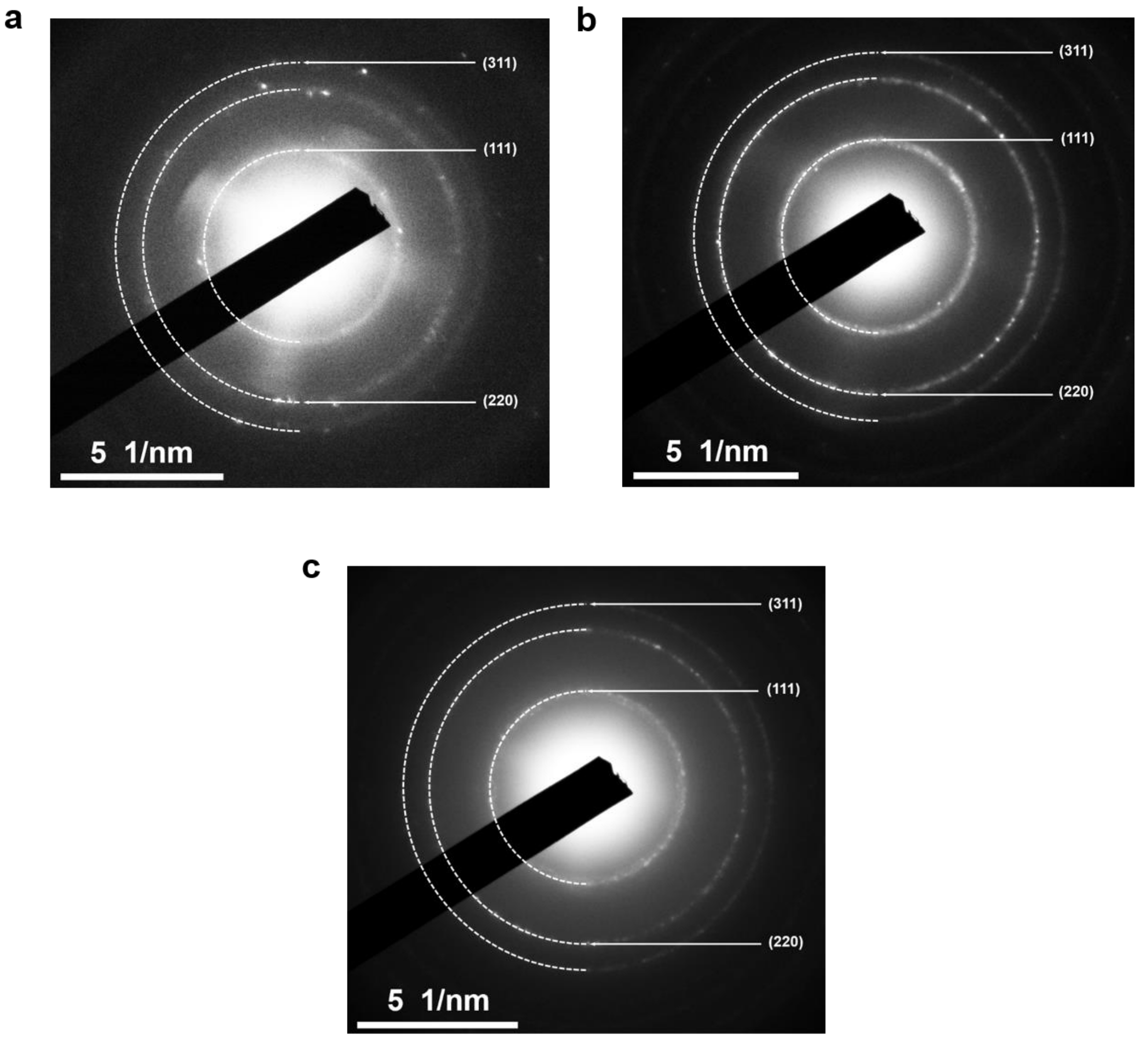

Figure S4. SAED patterns of (a) CdS#3, (b) CdS#6 and (c) CdS#9 QDs, which are consistent with the XRD patterns in Figure 1e.

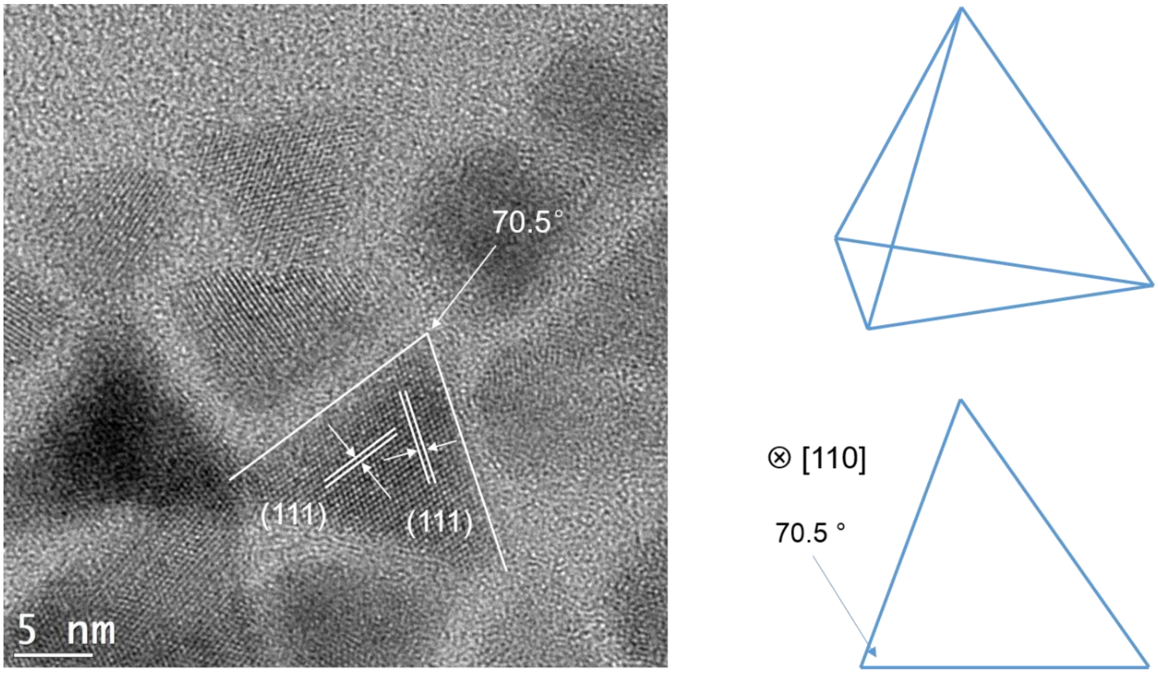

Figure S5. HRTEM image of CdS#9 g-QDs, showing two (111) facets and the angle of 70.5 degree observed from [110] direction, which is consistent with the three-dimensional pyramids geometry.

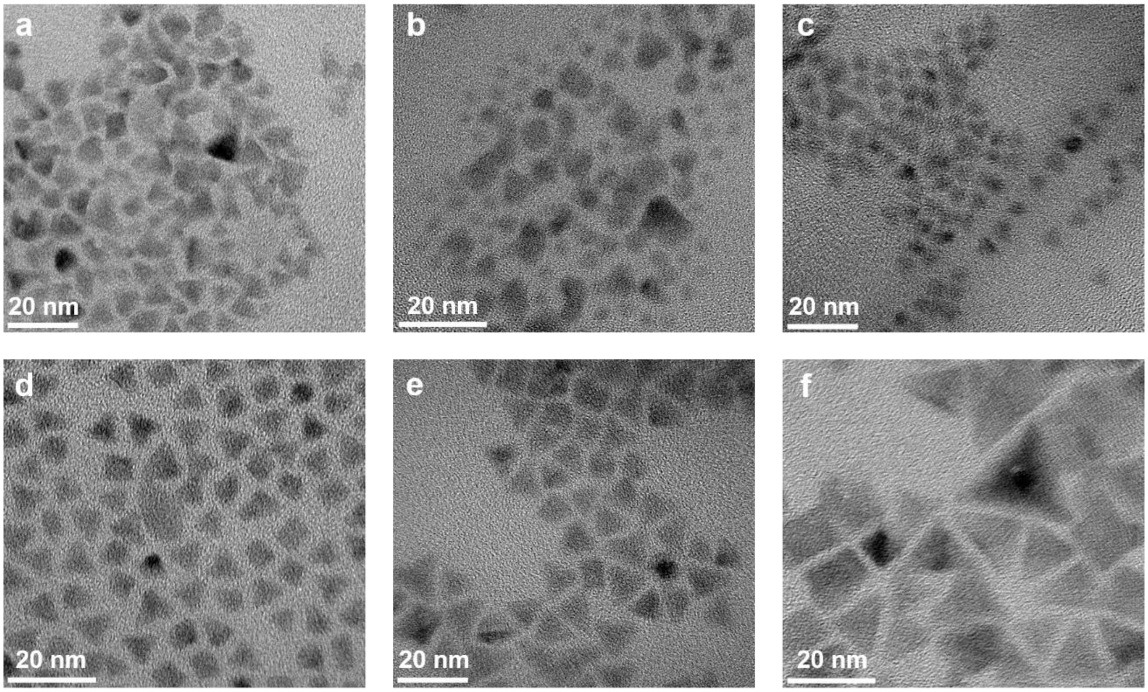

Figure S6. Representative TEM images of (a) CdS#1, (b) CdS#2, (c) CdS#4, (d) CdS#5, (e) CdS#7 and (f) CdS#8 QDs.

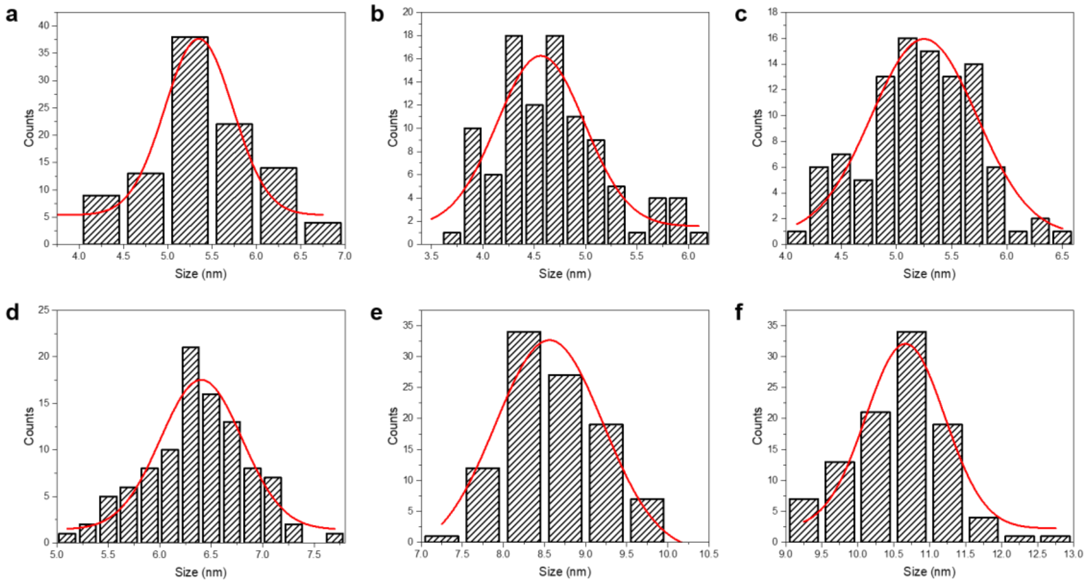

Figure S7. Size distribution of (a) CdS#1, (b) CdS#2, (c) CdS#4, (d) CdS#5, (e) CdS#7 and (f) CdS#8 QDs. Sizes were measured as the height of projected triangles [15] (distance from a vertex to the middle of the opposite side) in TEM images for at least 100 QDs.

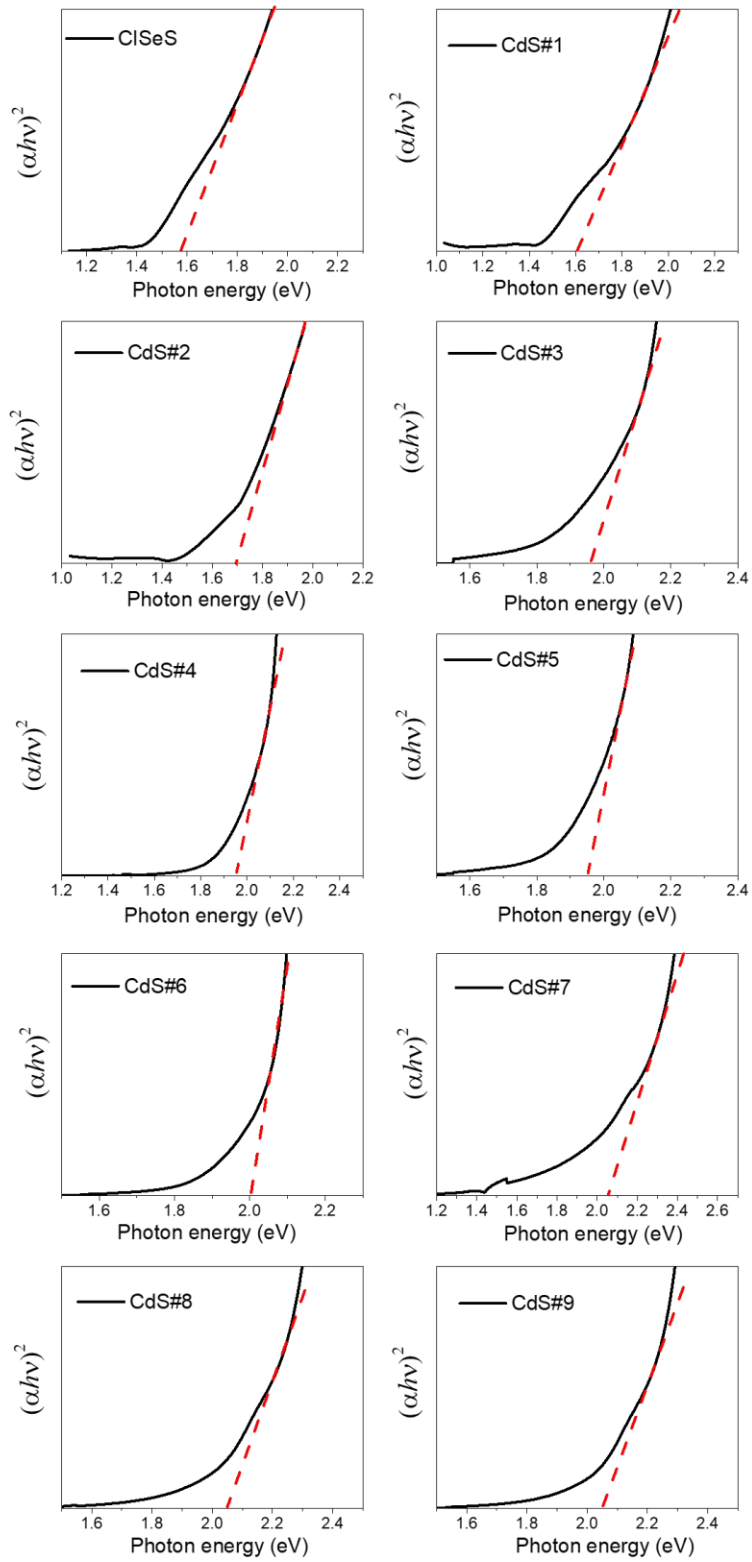

Figure S8.Tauc plot derived from absorption spectra of all QDs.

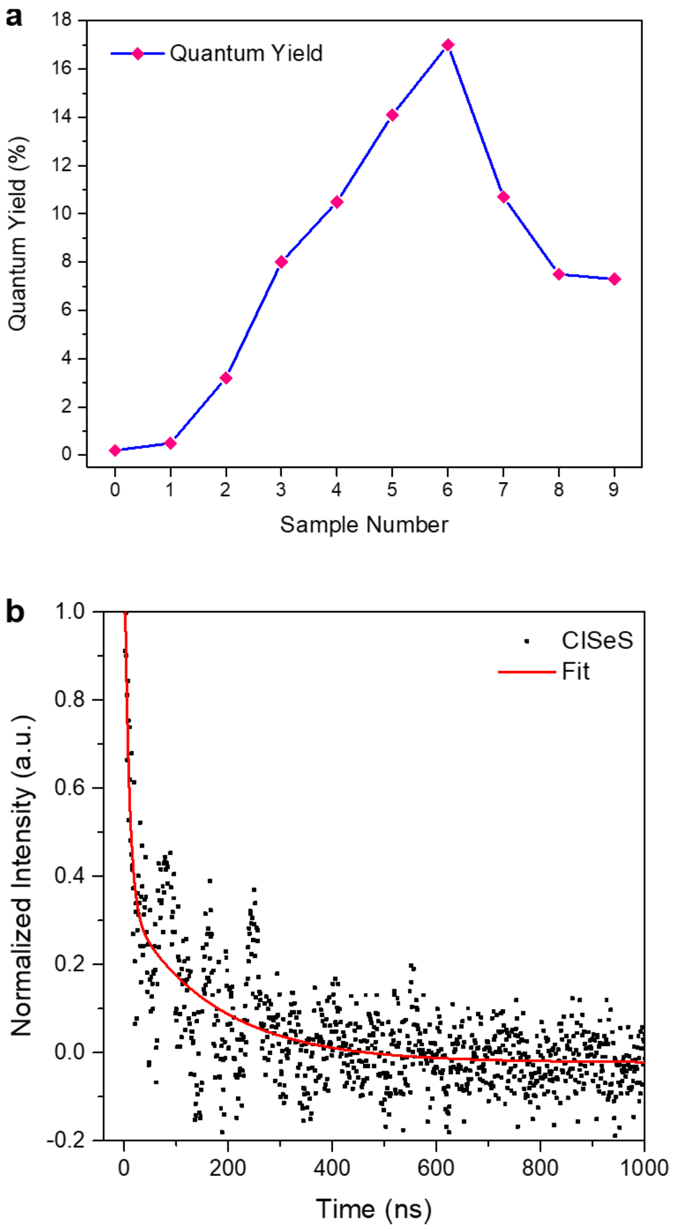

Figure S9. (a) Quantum yield of CISeS QDs (sample No. 0) and corresponding core/shell QDs (CdS#1 to CdS#9 QDs). (b) Transient PL spectrum of CISeS QDs with fitted average lifetime of ~165±4 ns.

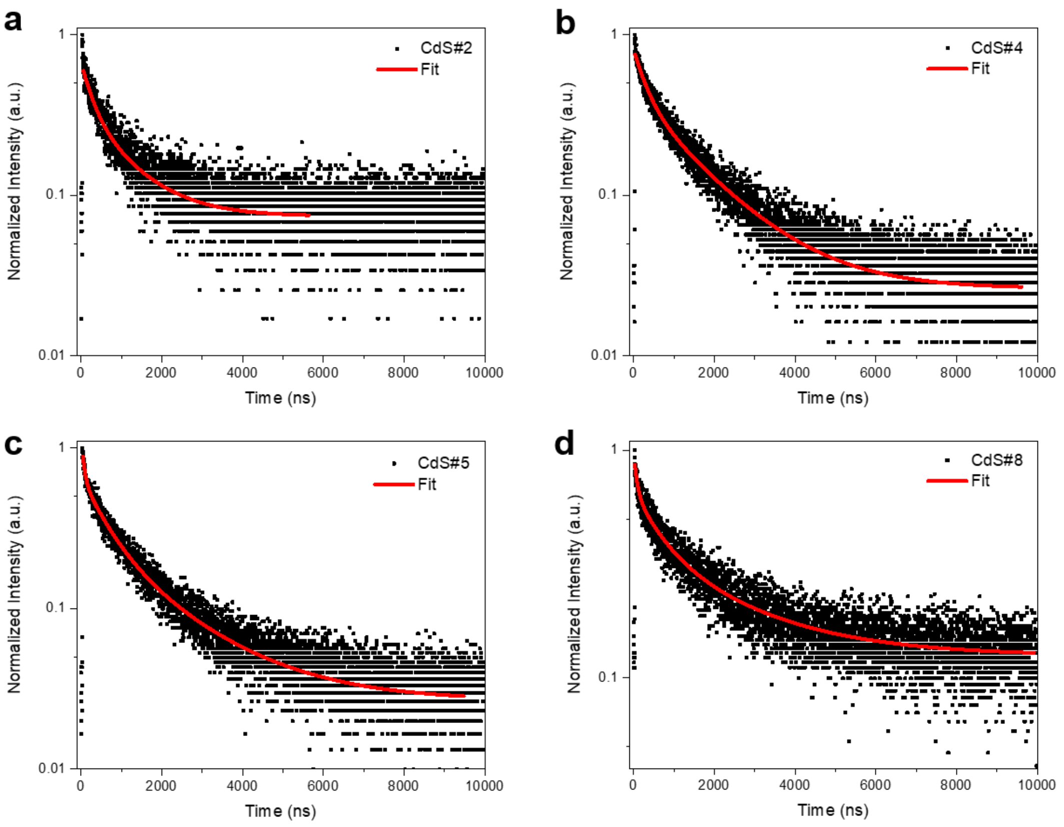

Figure S10. PL decay curves of (a) CdS#2, (b) CdS#4, (c) CdS#5 and (d) CdS#8 QDs, displaying fitted average lifetime of ~1.01 μs, 1.46 μs, 1.53 μs and 1.91 μs, respectively.

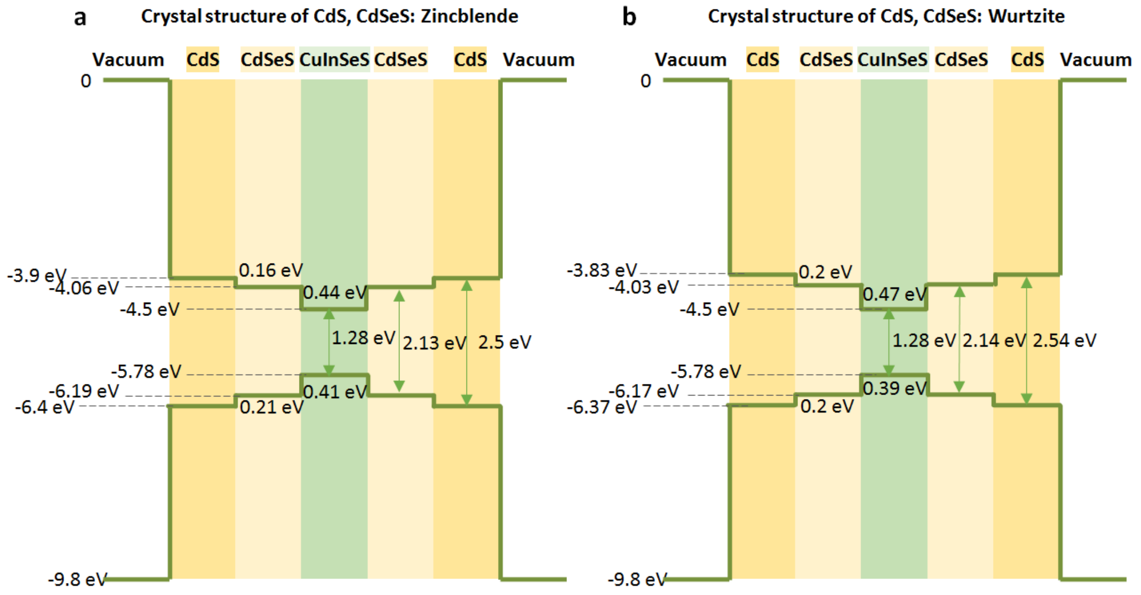

Figure S11. Band structure for the CISeS/CdSeS/CdS heterostructure. (a) The crystal structure of the shells (CdSeS and CdS) is ZB phase, which applies to the samples in the

experiments. (b) The crystal structure of the shells (CdSeS and CdS) is WZ phase, which are used for comparison purpose in theoretical models.

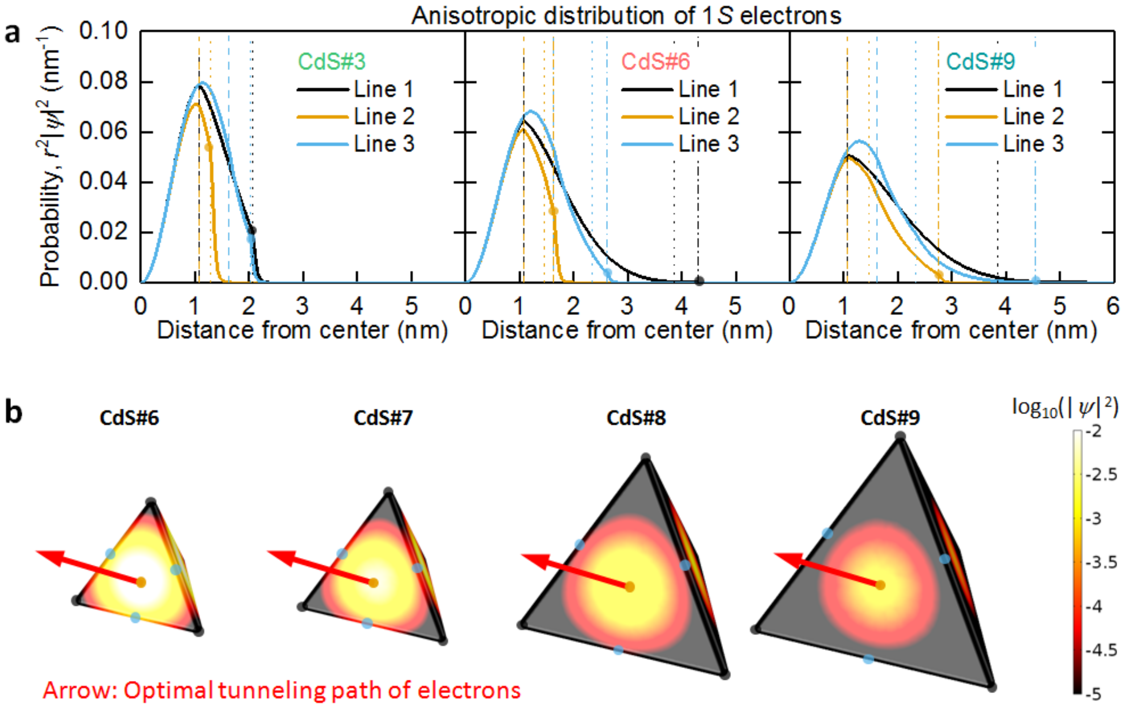

Figure S12. (a) Radial distributions of 1S electrons in the g-QDs (CdS#3, CdS#6, CdS#9) along three different directions. The Lines 1-3 are vectors pointing from the origin to the vertex, face center and edge center of the tetrahedron QD, respectively, as demonstrated in Figure 1b of the main text. (b) Maps of wave functions, $\log_{10}(|\psi|^2)$, at the QD surfaces for the g-QDs with two shells, CdS#6-9. The optical tunneling path of 1*S* electrons in the QDs is shown by the red arrow, which is along Line 2.

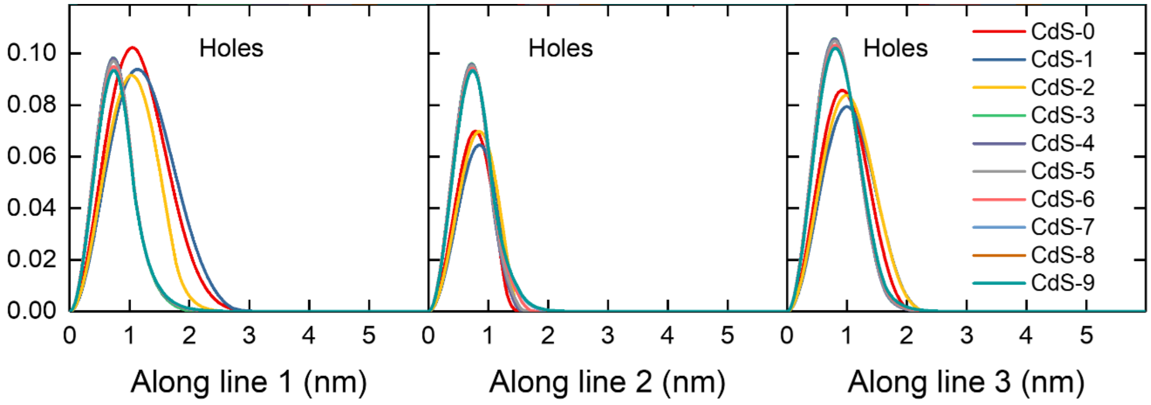

Figure S13. Normalized radial distribution function of holes in the series of g-QDs along Line 1, Line 2 and Line 3, respectively. The Lines 1-3 are vectors pointing from the origin to the vertex, face center and edge center of the tetrahedron QD, respectively.

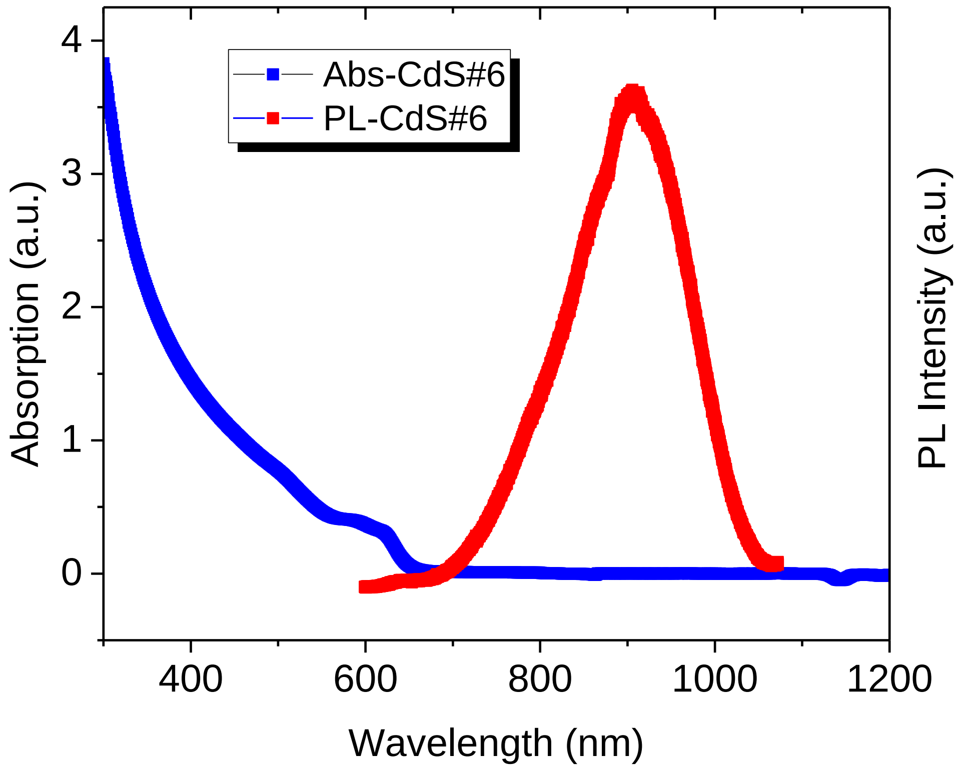

Figure S14. UV-visible absorption and PL spectra of optimized CdS#6 g-QDs for fabrication of PEC and solar cells.

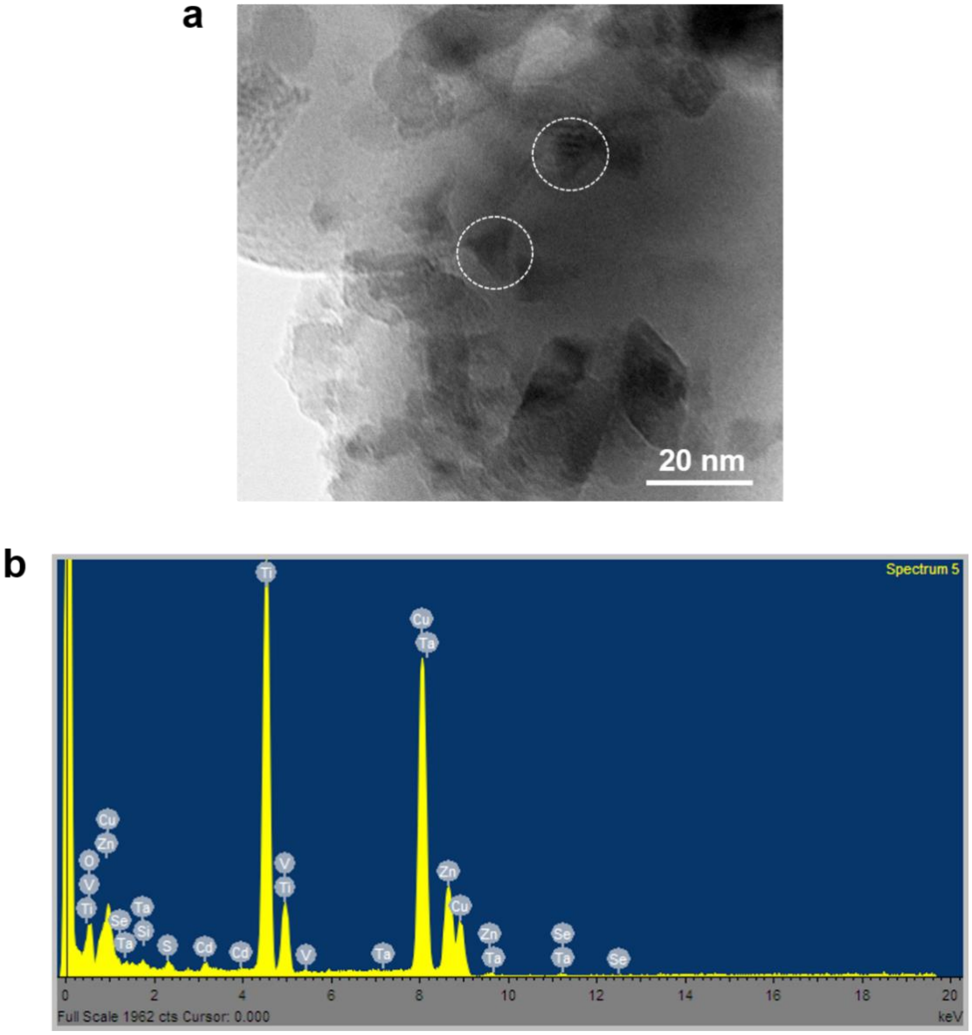

Figure S15. (a) TEM image of CdS#6 g-QDs-sensitized photoelectrode with homogeneous distribution of QDs (white dashed circle indicates the QDs on the surface of TiO_2 nanoparticles). (b) EDS spectra of CdS#6 g-QDs-sensitized photoelectrode.

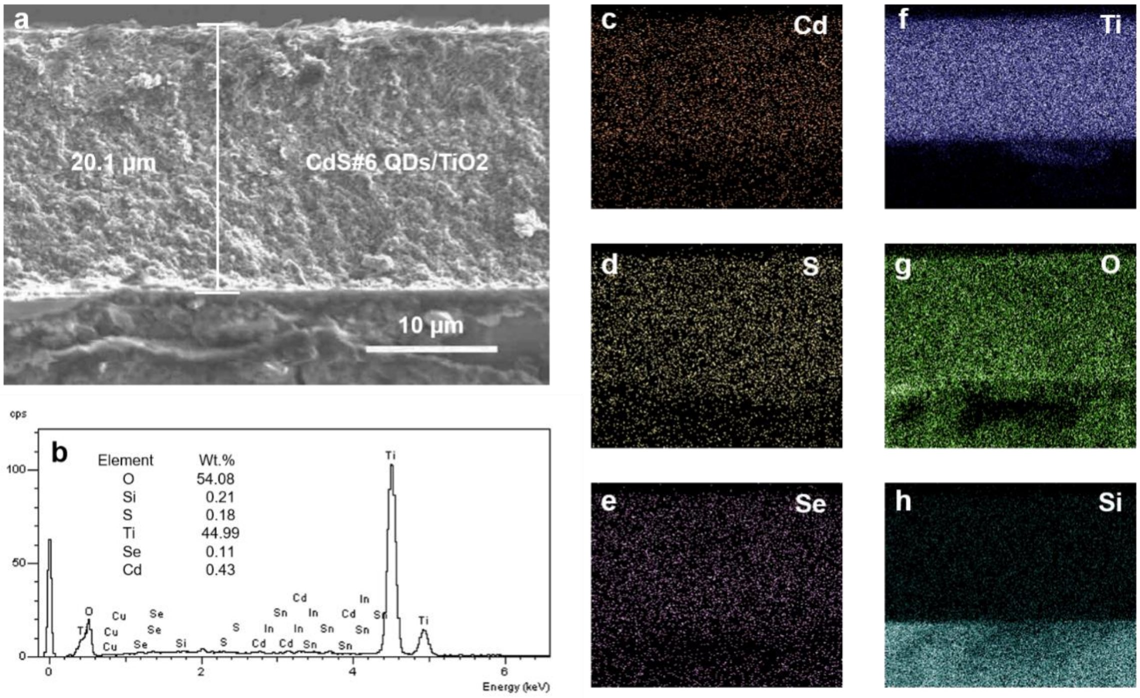

Figure S16. (a) Cross-sectional SEM image of CdS#6 g-QDs-sensitized photoanode and corresponding (b) EDS spectra. EDS mapping analysis of (c) Cd, (d) S, (e) Se, (f) Ti, (g) O and (h) Si.

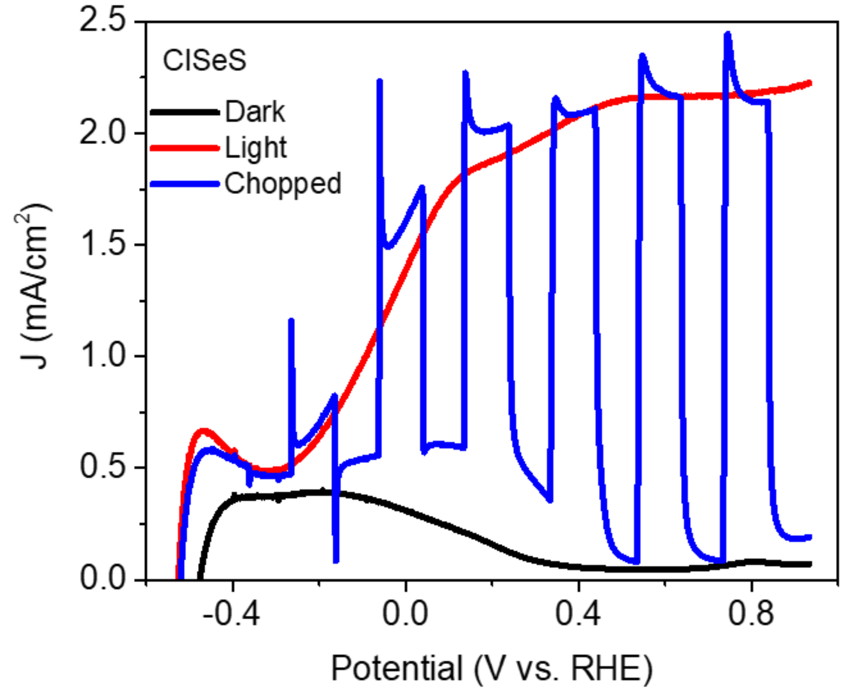

Figure S17. Linear sweep voltammetry of TiO_2/CISeS/ZnS photoanode in the dark (black curve), under continuous (red curve) and chopped (blue curve) under standard one sun illumination (AM 1.5 G, 100 mW/cm^2).

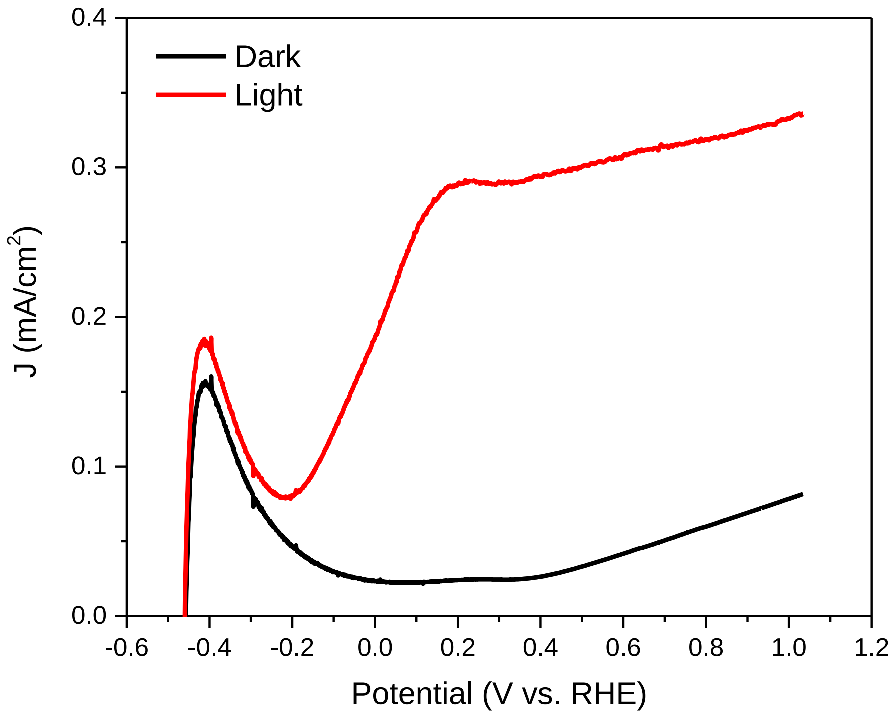

Figure S18. Linear sweep voltammetry of bare TiO_2-based photoanode in the dark and under AM 1.5 G irradiation at 100 mW/cm^2.

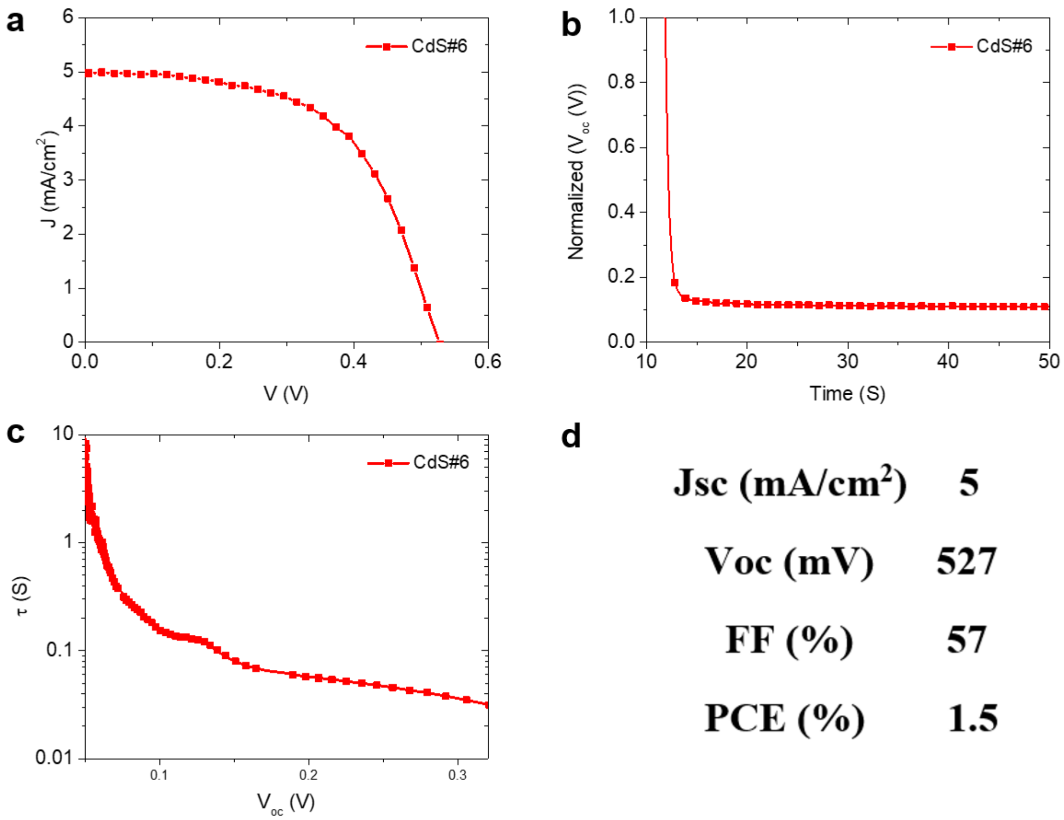

Figure S19. (a) Current density versus voltage curve of CdS#6 g-QDs based solar cells under one sun irradiation (AM 1.5 G, 100 mW cm^{-2}). (b) Open circuit voltage (V_{oc}) decay as the function of time. (c) Electron lifetime (τ) as a function of V_{oc} calculated from V_{oc} decay measurements. (d) Photovoltaic parameters calculated from I–V measurements of QDSCs based on CdS#6 g-QDs as light harvesters.
